# Integrated world modeling theory expanded: Implications for the future of consciousness

**DOI:** 10.3389/fncom.2022.642397

**Published:** 2022-11-24

**Authors:** Adam Safron

**Affiliations:** ^1^Department of Psychiatry and Behavioral Sciences, Johns Hopkins University School of Medicine, Center for Psychedelic and Consciousness Research, Baltimore, MD, United States; ^2^Cognitive Science Program, Indiana University, Bloomington, IN, United States; ^3^Institute for Advanced Consciousness Studies (IACS), Santa Monica, CA, United States

**Keywords:** consciousness, Integrated Information Theory (IIT), Global Neuronal Workspace Theory (GNWT), Free Energy Principle and Active Inference (FEP-AI) Framework, predictive turbo autoencoding, expander graphs, shared latent spaces, Graph Neural Networks (GNNs)

## Abstract

Integrated world modeling theory (IWMT) is a synthetic theory of consciousness that uses the free energy principle and active inference (FEP-AI) framework to combine insights from integrated information theory (IIT) and global neuronal workspace theory (GNWT). Here, I first review philosophical principles and neural systems contributing to IWMT’s integrative perspective. I then go on to describe predictive processing models of brains and their connections to machine learning architectures, with particular emphasis on autoencoders (perceptual and active inference), turbo-codes (establishment of shared latent spaces for multi-modal integration and inferential synergy), and graph neural networks (spatial and somatic modeling and control). Future directions for IIT and GNWT are considered by exploring ways in which modules and workspaces may be evaluated as both complexes of integrated information and arenas for iterated Bayesian model selection. Based on these considerations, I suggest novel ways in which integrated information might be estimated using concepts from probabilistic graphical models, flow networks, and game theory. Mechanistic and computational principles are also considered with respect to the ongoing debate between IIT and GNWT regarding the physical substrates of different kinds of conscious and unconscious phenomena. I further explore how these ideas might relate to the “Bayesian blur problem,” or how it is that a seemingly discrete experience can be generated from probabilistic modeling, with some consideration of analogies from quantum mechanics as potentially revealing different varieties of inferential dynamics. I go on to describe potential means of addressing critiques of causal structure theories based on network unfolding, and the seeming absurdity of conscious expander graphs (without cybernetic symbol grounding). Finally, I discuss future directions for work centered on attentional selection and the evolutionary origins of consciousness as facilitated “unlimited associative learning.” While not quite solving the Hard problem, this article expands on IWMT as a unifying model of consciousness and the potential future evolution of minds.

## Facing up to the enduring problems of consciousness with integrated world modeling theory

The Hard problem of consciousness asks, how can it be that there is “something that it is like” to be a physical system (Nagel, 1974; Chalmers, 1995)? The “meta-problem” of consciousness refers to the (potentially more tractable) challenge of addressing why it is that opinions and intuitions vary greatly with respect to what it would take to meaningfully answer this question (Chalmers, 2018). The “real problem” of consciousness refers to the further challenge of addressing why it is that different biophysical and computational phenomena correspond to different qualities of experience (Seth, 2016).

Integrated world modeling theory (IWMT) attempts to address these unsolved problems about the nature(s) of consciousness by combining Integrated Information Theory (IIT) and Global Neuronal Workspace Theory (GNWT) with the Free Energy Principle and Active Inference framework (FEP-AI). IIT speaks to the Hard problem by beginning from phenomenological axioms, and then goes on to postulate mechanisms that could realize such properties, ultimately coming to the conclusion that consciousness is “what physics feels like from the inside” (Koch, 2012). GNWT speaks to the real problem by focusing on the properties of computational systems that could realize the functions of consciousness as a means of globally integrating and broadcasting information from mental systems. FEP-AI has been used to address all these problems in a variety of ways, with IWMT representing one such attempt. For a detailed exploration of potential inter-relations between FEP-AI, IIT, and GNWT, please see the original publication of IWMT; for a high-level summary, please see Supplementary Appendix Figure A.

In attempting to explain how there could be “something that it *is like*” to be a physical system, it is worth noting that this question is often phrased as “something that it *feels like.*” The nature of embodied perception and affective states lie at the heart of what it would take to provide a satisfying solution to the Hard problem. Further, the Hard problem could be viewed as containing an implicit question: “something that it *feels like, for whom*?” While some may want to separate consciousness from sensations or selfhood (Tononi et al., 2016), it may also be the case that addressing the Hard problem requires understanding the nature of selves, and as Dennett (2018) has argued, “free will.” Along these lines, IWMT specifically places somatic experiences and agentic selfhood at the core of consciousness, and consciousness at the core of agency (Safron, 2021b).

Integrated world modeling theory specifically argues that integrated information and global workspaces only entail consciousness when applied to systems capable of functioning as Bayesian belief networks and cybernetic controllers for embodied agents (Seth, 2014; Safron, 2019, 2021b). That is, IWMT agrees with IIT and GNWT with respect to the integration and widespread availability of information as necessary preconditions for consciousness, but disagrees that these are sufficient enabling conditions for subjective experience. [Note: GNWT’s more specific claim is that workspaces help to select particular interpretations of events, which is highly compatible with IWMT, especially with more recent Bayesian interpretations of workspace dynamics (Mashour et al., 2020; Safron, 2020a; Whyte and Smith, 2020).] Rather, IWMT argues that *phenomenal consciousness is what integrated world-modeling is like, when generative processes are capable of jointly integrating information into models with coherence with respect to space (i.e., relative degrees of locality), time (i.e., relative changes within space), and cause (i.e., regularities with respect to these changes, potentially requiring some basic form of self/other-modeling) for systems and their relationships with their environments*. These coherence-making properties are stipulated to be required for situating modeled entities relative to each other with specific features, without which there would be no means of generating an experienceable world. Consciousness-entailing nervous systems (functioning as generative models) are stipulated to provide these sources of coherence via particular organizational features, as well as by having actual semantic content by virtue of evolving through interactions with a coherently structured (and so semi-predictable) world. IWMT further introduces a mechanism for generating complexes of integrated information and global workspaces via *self-organizing harmonic modes (SOHMs)*, wherein synchrony both emerges from and facilitates “communication-through-coherence” (Buzsáki and Watson, 2012; Fries, 2015; Deco and Kringelbach, 2016; Atasoy et al., 2018). SOHMs are proposed to both require and allow for high degrees of meaningful integrated information, where meaning is understood as differences that make a difference to the ability of systems to pursue their goals, including the goal of modeling the world for the sake of prediction and control.

Integrated world modeling theory focuses on the neural and computational bases of ‘basic’ phenomenal consciousness, but also has relevance for theories focused on “conscious access” and “higher order” knowledge, where some of these implications have been explored elsewhere with respect to goal-oriented behavior and cognition/affect (Safron, 2021b). However, while experience itself is proposed to be a unitary (and discrete) phenomenon, more abstract capacities for various forms of conscious access and self-awareness are considered to be more multifarious in their manifestations. These distinctions will be important for subsequent discussions in which we will consider the physical and computational substrates of consciousness adduced by various theories, where IWMT claims that multiple points of view may be valid with respect to issues as to whether experience is primarily realized by the frontal lobes or a “posterior hot zone” (Boly et al., 2017). Strangely, IWMT suggests that both these perspectives are likely accurate, but with respect to different explananda. That is, IWMT agrees with IIT that posterior cortices (and perhaps specific subnetworks thereof) provide necessary and sufficient conditions for realizing a consciousness-entailing generative (self-world) model over the sensorium of an embodied-embedded agent. Yet IWMT also agrees with GNWT that the frontal lobes are likely required for accessing such experiences in terms of being able to manipulate, reflect, and report on their contents (and contexts). However, IWMT also suggests that notions of conscious access may be insufficiently precise for progressive research and theory construction, in that by the time we are considering the processes contributing to such high-level functions, we may be forced to also consider ways in which cognition extends beyond brains and into bodies and extended embodiments/phenotypes, so cautioning against overly simple mappings between modeling and mechanisms. In what follows, we will explore the nature of these claims in greater depth than in the original publication, as well as additional considerations and future directions for understanding the nature of experience in biological and potentially artificial systems.

## Preconditions for experience: Space, time, cause, self, agency

By emphasizing the properties by which coherent world-modeling is made possible, the philosophical foundations of IWMT can be most strongly tied to the thought of Kant and Helmholtz. The core claims of the theory are particularly informed by Kant’s stipulation of synthetic *a priori* categories (i.e., complex concepts possessed in advance of experience) as preconditions for judgment. IWMT further argues that these preconditions for coherent knowledge are also preconditions for coherent experience, and focuses on the categories of space (i.e., relative localization of entities), time (i.e., relative transformations of entities in space), and cause (i.e., regularity with respect to transformations). Without spatial, temporal, and causal coherence, there can be no means of situating entities relative to each other with specific properties, and so there would be no means of generating an experienceable world. This position is consistent with both the axioms of IIT (e.g., composition), the kind of informational synergy emphasized by GNWT, and also the constructive epistemology of FEP-AI (Swanson, 2016). IWMT goes further in emphasizing the importance of selfhood, consistent with Kant’s notion of the transcendental unity of apperception in which spatiotemporal and causal information are bound together into a unified manifold via a unified experiencing subject (Northoff, 2012). While the stipulation of these properties of experience may help to address the question of why there may be “something that it feels like” to be some kinds of systems, a key question remains unanswered: to what degrees must these forms of coherence be present in which ways to enable different forms of consciousness? While this issue will not be definitively resolved here, we will consider neurophysiological and informational principles that may be illuminating.

Helmholtz extended Kant’s project in a more empirical direction, arguing that the experience of selfhood and freedom in willing are preconditions for deriving conceptions of space, time, and cause (De Kock, 2016). According to Helmholtz, a self/world distinction and sense of agency are both required for making sense of sensory observations, including with respect to constructing these categories of experience. This more empirically focused perspective is contrasted with Liebnizian (Sleigh, 2003) notions of “pre-established harmony” as an explanation for how minds come to be equipped with precisely the intuitions required for making sense of the world. In this way, Helmholtz rejected the *a priori* status of Kantian categories as part of his general project of deflating mysticism, which elsewhere involved critiquing the vitalist posit of a supernatural force animating living things (i.e., *élan vital*). IWMT was developed in the same spirit as Helmholtz’s naturalization of mind and nature, although with somewhat greater sympathies to notions of pre-established harmonies, since evolution by natural selection represents a means by which mental systems could come to non-mystically resonate with essential properties of the world (Ramstead et al., 2017; Badcock et al., 2019; Zador, 2019).

Helmholtz’s argument for selfhood and agency as foundational cognitive capacities is fully compatible with IWMT and FEP-AI. The necessity of autonomy for coherent modeling is emphasized in FEP-AI, in which expected free energy (i.e., precision-weighted cumulative prediction errors with respect to preferred states) is minimized via action/policy selection over predictive models for future (counterfactual) goal realization (Friston et al., 2017a; Friston, 2018). In these ways, IWMT supports both Kantian and Helmholtzian views on the preconditions and origins of mind. IWMT also agrees with Kant’s view in that the process of bootstrapping minds (Gentner, 2010; Tenenbaum et al., 2011; Safron, 2021b) likely requires some pre-established modes of cognitive organization (Spelke and Kinzler, 2007). For example the place/grid cells of the hippocampal/entorhinal system could contribute initial structuring of experience according to space and time (Moser et al., 2008; Buzsáki and Tingley, 2018)—although these response-properties may substantially depend on experience for their emergence (Kropff and Treves, 2008; Kaplan and Friston, 2018)—with a general capacity for tracking time-varying sequences being a potentially ubiquitous feature of cortex (Hawkins and Blakeslee, 2004). Implicit objective functions from innate salience mechanisms—e.g., maximizing information gain and empowerment (Redgrave et al., 2008; de Abril and Kanai, 2018)—and neuroplasticity rules such as spike-timing dependent plasticity (Hayek, 1952; Markram et al., 2011) could both be thought of as “evolutionary priors” that further help to organize experience according to likely patterns of causal influence (e.g., causes ought to precede effects). However, Helmholtz’s criticism of Kant’s intuitions may also highlight important differences between initial inductive biases and later constructive modeling of space (Terekhov and O’Regan, 2016), time (Buonomano, 2017; Wittmann, 2017), and cause (Buchsbaum et al., 2012). It may be misleading to refer to largely innate mechanisms for structuring experience as “intuitions,” as these capacities may lack experiential content by not (yet) affording sufficient coherence for the generation of an experienceable world. Finally, agency-related knowledge may be particularly complex, diverse in its forms, and dependent upon experience for its development (Kushnir et al., 2015; Kushnir, 2018; Chernyak et al., 2019).

Hence, while IWMT suggests that quasi-Kantian categories may represent essential “core knowledge” for bringing forth a world with particular properties (such that they may be experienced), many questions remain unanswered. To what extent are our intuitions of space and time elaborated by our intuitions regarding causal unfolding that depend on the agentic self as a point of view on the world (De Kock, 2016; Ismael, 2016)? If coherence-making is bidirectional in this way, would this imply a kind of mutual bootstrapping in learning of self, agency, and space/time/cause over the course of development? If sense-making involves this kind of bidirectionally, or capacity for inferential positive feedback, could the mutual dependency of subjective categories of experience partially explain non-linear shifts in psychological development (Isler et al., 2018)? Do different categories and intuitions asymmetrically drive different parts of development at different points in time? While these questions will not be definitively answered here, they may point the way to helping to identify which systems possess which forms of consciousness.

## Neural systems for coherent world modeling

As hopefully is made clear by the preceding discussion, philosophical considerations may be invaluable for helping to identify fundamental properties enabling conscious experience. Whether considered as synthetic *a priori* categories or experience-dependent constructed intuitions, the foundations of mind suggest that a primary task for cognitive science should be characterizing these properties on functional, algorithmic, and implementational levels of description. While such an analysis is beyond the scope of a single article, here I suggest neural systems that could contribute to some of these foundational capacities.

**FIGURE 1:**
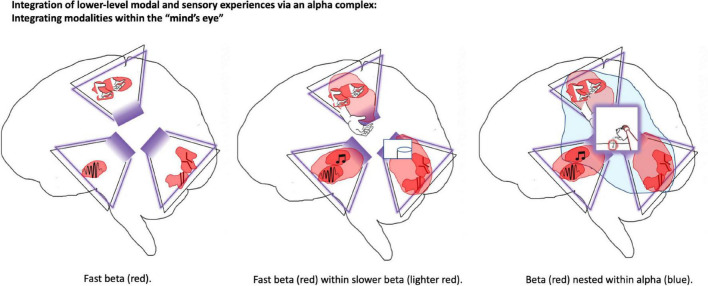
Precuneus as shared latent (work) space and source of visuospatial phenomenology. This figure depicts elements of world-modeling within the brain of a person who is pouring themselves a cup of tea. The precuneus may be particularly central for integrated world modeling. This posterior-medial structure is depicted as a kind of “Cartesian theater” that provides a basis for visuospatial modeling and phenomenal experience. In IWMT, “self-organizing harmonic modes” (SOHMs) are introduced as mechanisms in which synchronous complexes provide enhanced communication-through-coherence, entailing the calculation of joint marginal probability distributions for the subnetworks over which they form. This image depicts SOHMs in the form of beta complexes (in shades of red) and an alpha complex (in blue). Trapezoid-like shapes outlined in purple represent folded (recurrent) autoencoders, which provide an algorithmic description of the kinds of computation realized by these biophysical processes. (For more information about autoencoders, please see Supplementary Appendix; Safron, 2020a). In the left panel, small and fast beta-synchronized SOHMs close to primary modalities infer estimates of the causes of sensations in terms of low-level stimulus features. In the middle panel, these features are combined within the synchronization manifold provided by somewhat larger and slower forming beta SOHMs, so providing a source of more abstract and potentially more behaviorally meaningful object-like modeling. In the right panel, SOHMs evolving at alpha/beta-frequencies aggregate information for precuneus-centered models in which complex features are bound together into an even larger visual field with specific composition and integrated information content. IWMT suggests that this is the level of deep temporal modeling at which visuospatial consciousness is achieved, and also explicit re(–)presentations. While not depicted, a similar three-level hierarchy may be involved with the generation of somatospatial awareness from lateral parietal cortices. These shared latent (work)spaces for autoencoding hierarchies are suggested to be structured according to the principles of geometric deep learning as kinds of graph neural networks. Taken together, the “mind’s eye” and “lived body” (whose coupling may potentially be mediated by an additional graph-mesh neural network for attention/intention schemas) would constitute the physical and computational substrates for phenomenal consciousness, functioning as an integrated generative world model and cybernetic controller for embodied-embedded agents. Perhaps strangely, experience may be exclusively realized via visuospatial and somatospatial awareness, including with respect to seemingly non-spatial/somatic modalities such as hearing and olfaction.

Integrated world modeling theory identifies two main sources of consciousness for space: (1) a sense of locality based on body-centric coordinates (Terekhov and O’Regan, 2013), and (2) introspectable 2D maps (Haun and Tononi, 2019) organized according to quasi-Cartesian coordinates with irregular spacing biased by salience and ‘navigation’ potential. Body-centric spatial senses would likely primarily be found in superior and inferior parietal cortices based on convergence of the dorsal visual stream and upper levels of the somatosensory hierarchy. 2D spatial maps can be found throughout the brain, but consciously accessible mappings are likely primarily localized to the precuneus at the brain’s posterior midline. These precuneus-based maps may couple with the more well-known spatial maps of the hippocampal/entorhinal system (Moser et al., 2008; Faul et al., 2020), so allowing for ‘navigating’ (Kaplan and Friston, 2018) through visualized domains. IWMT suggests that hippocampal representations of spatiotemporal trajectories are unlikely to be directly introspectable, as deep spatiotemporal hierarchies and grounding within sensory modalities are likely required for coherent conscious experience. Precuneus-based maps may also be aligned with dynamics in the dorsomedial prefrontal cortex (another midline structure) (Hassabis et al., 2014; Li et al., 2018; Faul et al., 2020), which may potentially be interpreted as sources of “attention schemas” (Graziano, 2019), upper levels of action hierarchies, and—perhaps most saliently with respect to conscious awareness—as an additional level of hierarchical control over the pre-supplementary eye fields (Safron, 2021b). With precise sequencing shaped by striatal-thalamic-cerebellar loops (Gao et al., 2018), these frontal representations may provide a source of coherent vectors for directing the “mind’s eye,” so influencing what is likely to be ‘projected’ onto the precuneus as a kind of inner ‘theater’ (Figure 1). Mechanistically, these action-oriented influences on perception may further depend on pulvinar-mediated synchrony for their realization (O’Reilly et al., 2017; Hu et al., 2019).

Integrated world modeling theory suggests that we ought to expect all phenomenal content to involve spatial aspects, potentially requiring multi-level processes of spatialization. Indeed, we may parse complex features by performing a kind of multidimensional scaling (Hout et al., 2013) in which features are mapped onto 2D spaces. The hippocampal/entorhinal system may be particularly important for establishing these mappings (Bellmund et al., 2016, 2018; Nau et al., 2018), and potentially for establishing the routes by which we are able to make sense of these complex domains by performing (generalized) ‘navigation’ through their spatialized representations (Safron et al., 2021a). For example, it has recently been demonstrated that entorhinal grid cells are used to spatially organize reward-related representations in the ventromedial prefrontal cortex (another midline region), with spatialization of task structure having behavioral significance for reinforcement learning problems (Baram et al., 2019).

The nature of time perception may be somewhat more complicated compared to space, and may even be conceptually derived from initially spatial understanding (Levin et al., 1978; Levin, 1992). While the entire brain (or at least much of the neocortex) may be sensitive to temporally varying sequences (Hawkins and Blakeslee, 2004), there seems to be no singular clock for time perception. One candidate clock-like mechanism potentially includes the formation of “global emotional moments” via the insular salience hierarchy (Craig, 2009), with a greater density of salient events corresponding to relatively slower experienced (but not necessarily remembered) temporal unfolding. Speculatively, dopaminergic influences on time perception (Soares et al., 2016; Buonomano, 2017) may suggest that the ability to both track and simulate (and track via simulations) causal sequences via actions may provide another factor influencing time perception, with a greater frequency of actions corresponding to elongated subjective timelines. Non-mutually exclusively, relationships between dopamine and time perception could be mediated by the hippocampal/entorhinal system (Mannella et al., 2013; McNamara and Dupret, 2017). These influences could include multiple factors, such as the frequency with which events are encoded as new memories, or through the mapping of timelines onto (2D) spatial trajectories with place/grid cells. Indeed, abilities to construct maps and routes for navigation (broadly construed) may be primary means by which space and time come together in brain and mind. Such simultaneous localization and mapping mechanisms may provide a basis for both the spatialization of time as well as the temporalization of space, as these two modes of quantization are fundamentally linked (and mutually defined) in terms of velocity, which may be physiologically linked via locomotion-dependent cholinergic midbrain nuclei (Lee et al., 2014). Velocity estimation both requires and enables the ability to track changing relative spatial locations, with speed being time-varying displacement within space. Speculatively, similar relationships between time and space might also be mediated by mapping events onto body maps, both in terms of using bodily representations as a kind of space (within which things can change at varying speeds), as well as via potential magnitude estimation via the intensity of proprioceptive and interoceptive sensations. Finally, for linguistic beings such as humans, it may be difficult to overstate the importance of analogical/metaphorical construction processes for tying together and expanding these fundamental categories (Jaynes, 1976; Lakoff and Johnson, 1999; Safron, 2019).

Causal understandings may be more difficult to map unto neural systems than time and space. As previously mentioned, some proto-causal understanding may derive from mechanisms such as the ability of spike-timing dependent plasticity to arrange events into likely time-varying sequences (Hayek, 1952; Markram et al., 2011)—wherein causes can be expected to precede events—or via salience mechanisms such as modulation of midbrain dopamine by whether events are likely to have been internally or externally generated (Redgrave et al., 2008; de Abril and Kanai, 2018). However, understanding causation requires more than these proto-intuitions, and in particular the ability to generate counterfactual scenarios involving simulated interventions, potentially providing an implementation of the “do-operator” introduced by Judea Pearl for causal inference with graphical models (Pearl and Mackenzie, 2018). While it is unclear whether anything like the graphical representations underlying Pearlean analysis are used by brains and minds, the ability to simulate a variety of actions/interventions could provide a basis for similar kinds of causal reasoning. However, this ability to generate counterfactual scenarios likely required the advent of internal dynamics that can be decoupled from immediate engagement with the environment. Intriguingly, such adaptations may have arisen relatively straightforwardly with increasing degrees of cortical expansion, some of which may have provided a difference in kind with respect to expanded association cortices and a more freely operating default mode network (Buckner and Krienen, 2013; Sormaz et al., 2018).

Finally, while the potential complexities of selfhood are inexhaustible, a very minimal sense of self and agency could potentially be derived from the reliable ability of embodied brains to learn that bodies depend on particular sensors by which they can perceive and effectors by which they can act. Since sensors and effectors are located on and in the body—and not elsewhere—the fact that bodily states are uniquely perceivable and controllable may provide a relatively straightforward means of construing models in which an agentic self exists as a separate entity from the rest of the (less immediately perceivable/controllable) world. While a broad range of neural systems may contribute to self-consciousness in diverse ways, IWMT focuses on body maps and visuospatial models for scaffolding inferences about selves and the (life)worlds in which they find themselves embedded.

## Machine learning architectures and predictive processing models of brain and mind

Integrated world modeling theory suggests that many of the processes and systems underlying consciousness may also be describable in terms of computational principles from machine learning. It may seem rather implausible that present technologies could reveal deep principles about the nature of mind, with potentially cautionary tales to be found in previous metaphorizations based on the technology of the day. Is this just another case of naïve arrogance of overgeneralizing from the familiar and fashionable, akin to previous claims that minds could be understood in terms of the accumulation and release of pressures, or when nervous systems were suggested to function according to the logical operations found in computers (McCulloch and Pitts, 1943)? Metaphors in which brains are understood as computers and even steam engines are both consistent with IWMT and the Free Energy Principle and Active Inference (FEP-AI) framework. Not only is there necessarily a sense in which brains compute information, but the serial operation of conscious access may even be thought of as a kind of (neural) Turing machine (Dehaene, 2014; Graves et al., 2014). Even more, if neural systems minimize [informational (and possibly thermodynamic)] free energy (Kiefer, 2020), then this may not only provide computational justification for pressure-based analogies (Carhart-Harris and Friston, 2010), but potentially even models inspired by the causal powers of engines as systems that perform thermodynamic work cycles (Safron, 2020a,2021b). Thus, these previous attempts to analogize the nature of mind with existing technologies may have been surprisingly prescient.

**FIGURE 2:**
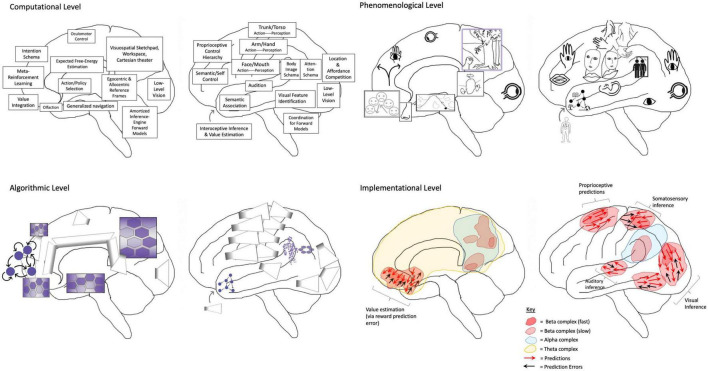
Depiction of the human brain in terms of phenomenological correspondences, as well as computational (or functional), algorithmic, and implementational levels of analysis (Reprinted from Safron, 2021b). Depiction of the human brain in terms of entailed aspects of experience (i.e., phenomenology), as well as computational (or functional), algorithmic, and implementational levels of analysis (Marr, 1983; Safron, 2020b). A phenomenological level is specified to provide mappings between consciousness and these complementary/supervenient levels of analysis. Modal depictions connotate the radically embodied nature of mind, but not all images are meant to indicate conscious experiences. Phenomenal consciousness may solely be generated by hierarchies centered on posterior medial cortex, supramarginal gyrus, and angular gyrus as respective visuospatial (cf. consciousness as projective geometric modeling) (Rudrauf et al., 2017; Williford et al., 2018), somatic (cf. grounded cognition and intermediate level theory) (Varela et al., 1992; Barsalou, 2010; Prinz, 2017), and intentional/attentional phenomenology (cf. Attention Schema Theory) (Graziano, 2019). Computationally, various brain functions are identified according to particular modal aspects, either with respect to generating perception (both unconscious and conscious) or action (both unconscious and potentially conscious, via posterior generative models). [Note: Action selection can also occur via affordance competition in posterior cortices (Cisek, 2007), and frontal generative models could be interpreted as a kind of forward-looking (unconscious) perception, made conscious as imaginings via parameterizing the inversion of posterior generative models]. On the algorithmic level, these functions are mapped onto variants of machine learning architectures—e.g., autoencoders and generative adversarial networks, graph neural networks (GNNs), recurrent reservoirs and liquid state machines—organized according to potential realization by neural systems. GNN-structured latent spaces are suggested as a potentially important architectural principle (Zhou et al., 2019), largely due to efficiency for emulating physical processes (Battaglia et al., 2018; Bapst et al., 2020; Cranmer et al., 2020). Hexagonally organized grid graph GNNs are depicted in posterior medial cortices as contributing to quasi-Cartesian spatial modeling (and potentially experience) (Haun and Tononi, 2019; Haun, 2020), as well as in dorsomedial, and ventromedial PFCs for agentic control. With respect to AI systems, such representations could be used to implement not just modeling of external spaces, but of consciousness as internal space (or blackboard), which could potentially be leveraged for reasoning processes with correspondences to category theory, analogy making via structured representations, and possibly causal inference. Neuroimaging evidence suggests these grids may be dynamically coupled in various ways (Faul et al., 2020), contributing to higher-order cognition as a kind of navigation/search process through generalized space (Hills et al., 2010; Kaplan and Friston, 2018; Çatal et al., 2021). A further GNN is speculatively adduced to reside in supramarginal gyrus as a mesh grid placed on top of a transformed representation of the primary sensorimotor homunculus (cf. body image/schema for the sake of efficient motor control/inference). This quasi-homuncular GNN may have some scaled correspondence to embodiment as felt from within, potentially morphed/re-represented to better correspond with externally viewed embodiments (potentially both resulting from and enabling “mirroring” with other agents for coordination and inference) (Rochat, 2010). Speculatively, this partial translation into a quasi-Cartesian reference frame may provide more effective couplings (or information-sharing) with semi-topographically organized representations in posterior medial cortices. Angular gyrus is depicted as containing a ring-shaped GNN to reflect a further level of abstraction and hierarchical control over action-oriented body schemas—which may potentially mediate coherent functional couplings between the “lived body” and the “mind’s eye”—functionally entailing vectors/tensors over attentional (and potentially intentional) processes (Graziano, 2018). Frontal homologs to posterior GNNs are also depicted, which may provide a variety of higher-order modeling abilities, including epistemic access for extended/distributed self-processes and intentional control mechanisms. These higher-order functionalities may be achieved via frontal cortices being more capable of temporally extended generative modeling (Parr et al., 2019c), and potentially also by virtue of being located further from primary sensory cortices, so affording (“counterfactually rich”) dynamics that are more decoupled from immediate sensorimotor contingencies. Further, these frontal control hierarchies afford multi-scale goal-oriented behavior via bidirectional effective connectivity with the basal ganglia (i.e., winner-take-all dynamics and facilitation of sequential operations) and canalization via diffuse neuro-modulator nuclei of the brainstem (i.e., implicit policies and value signals) (Houk et al., 2007; Humphries and Prescott, 2010; Stephenson-Jones et al., 2011; Dabney et al., 2020; Morrens et al., 2020). Finally, the frontal pole is described as a highly non-linear recurrent system capable of shaping overall activity via bifurcating capacities (Tani, 2016; Wang et al., 2018)—with potentially astronomical combinatorics—providing sources of novelty and rapid adaptation via situation-specific attractor dynamics. While the modal character of prefrontal computation is depicted at the phenomenological level of analysis, IWMT proposes frontal cortices might only indirectly contribute to consciousness via influencing dynamics in posterior cortices. Speculatively, functional analogs for ring-shaped GNN salience/relevance maps may potentially be found in the central complexes of insects and the tectums of all vertebrates (Honkanen et al., 2019), although it is unclear whether those structures would be associated with any kind of subjective experience. Even more speculatively, if these functional mappings were realized in a human-mimetic, neuromorphic AI, then it may have both flexible general intelligence and consciousness. In this way, this figure is a sort of pseudocode for (partially human-interpretable) AGI with “System 2” capacities (Bengio, 2017; Thomas et al., 2018), and possibly also phenomenal consciousness. [Note: The language of predictive processing provides bridges between implementational and computational (and also phenomenological) levels, but descriptions such as vector fields and attracting manifolds could have alternatively been used to remain agnostic as to which implicit algorithms might be entailed by physical dynamics]. On the implementational level, biological realizations of algorithmic processes are depicted as corresponding to flows of activity and interactions between neuronal populations, canalized by the formation of metastable synchronous complexes (i.e., “self-organizing harmonic modes”; Safron, 2020a). [Note: The other models discussed in this manuscript do not depend on the accuracy of these putative mappings, nor the hypothesized mechanisms of centralized homunculi and “Cartesian theaters” with semi-topographic correspondences with phenomenology].

Considering that FEP-AI has foundations in the free-energy objective functions used to train Helmholtz machines and autoencoders (Dayan et al., 1995), the rise of deep learning may have afforded conceptual progress for understanding not just minds, but all dynamical systems (viewed as generative models). The idea that deep learning could potentially inform neuroscience ought to be relatively unsurprising (Hassabis et al., 2017; Richards et al., 2019), in that artificial neural networks were designed to try to capture relevant aspects of nervous systems (McCulloch and Pitts, 1943; Lecun et al., 1998), albeit with limited physiological detail and some biologically implausible functionalities (e.g., training by backpropagation). IWMT goes further in arguing that not only can useful computational principles be derived from machine learning, but some architectures may have close correspondences with the neural processes contributing to consciousness via coherent world modeling. Below I will review a few of these relevant technologies and the ways functionally equivalent processes might be realized in biological systems (Figure 2). (For more detailed illustrations of these putative functional mappings, please see Supplementary Appendix Figures B, C). I will then go on to consider the implications of these suggested computational mappings for informing IWMT and associated theories.

### Cortex as folded disentangled variational autoencoder heterarchy

A predictive coding model of cortex may be approximated by folding a disentangled variational autoencoder over at the low-dimensional bottleneck such that levels align in encoders and generative decoders (please see Supplementary Appendix, “Autoencoders,” as well as Supplementary Appendix Figure B), respectively implemented via hierarchies of superficial and deep pyramidal neurons. To implement predictive coding, descending messages from generative decoder networks would continuously suppress (or “explain away”) ascending messages from encoders. In this coding scheme, only failed predictions from generative decoders get passed upwards through encoders, with these prediction errors continuing to rise up hierarchical levels until they can be successfully suppressed by the descending stream. These descending predictions are generated on multiple levels, both locally via recurrent dynamics, as well as on a more global basis, potentially accompanied by unique architectural features and discrete updating of integrative models (Friston et al., 2017b; Parr and Friston, 2018b). Viewed as folded autoencoders, these higher-level predictions would constitute a parameterization of generative decoder networks by samples from reduced-dimensionality latent feature spaces. As training proceeds, such an architecture should form increasingly predictive and sparse representations, so maximizing inferential power, while also minimizing the number of messages that need to be passed. This training for prediction and sparsification would correspond to the development of models of increasing accuracy, efficiency, and robust generalizability (Srivastava et al., 2014; Ahmad and Scheinkman, 2019).

A predictive coding model of cortex would correspond to not just a single (folded) autoencoder hierarchy, but a heterarchy composed of multiple intersecting hierarchies, so enabling cortical learning systems to obtain inferential synergy through multi-modal sensory integration (McGurk and MacDonald, 1976; Eguchi et al., 2020). In terms of machine learning principles, high-bandwidth connections between association cortices could correspond to the chaining of low-dimensionality bottlenecks from multiple autoencoders, so forming an auto-associative network capable of supporting loopy belief propagation (the potential functional significance of which will be explored below). Neuroanatomically speaking, these highly connected areas would correspond to the brain’s “rich club” networks (Heuvel et al., 2012), including the 2D grid structures described above (Figure 1), which could contribute to spatiotemporal modeling (Haun and Tononi, 2019) in both concrete physical and abstract (via spatialization) domains.

Theoretically, these subnetworks (entailing shared latent space) may be well-modeled as graph neural networks (GNNs) (Zhou et al., 2019; Safron, 2020a,b), which are gaining increasing popularity as a means of efficiently modeling a broad range of processes. From this perspective, experience-dependent plasticity may be understood as implementing a kind of implicit neural architecture search, which may potentially produce GNN-like representational structures as means of ensuring sufficiently rapid inference that estimates of system-world configurations are capable of both informing and being informed by action-perception cycles for embodied-embedded agents. Yet it remains unclear whether inferences from these subnetworks would themselves represent the physical/computational substrates of consciousness, or whether they would rather be necessary (but not sufficient) conditions for realizing phenomenality (Safron, 2021c). While this is not a necessary entailment of IWMT (and hence not a condition for falsification), if deep association cortices were found to operate according to principles of geometric deep learning, then it would provide strong support for the ideas presented here.

Finally, the regulation of neuronal dynamics by diffuse neuromodulator systems could be computationally understood as parameterizing inference and learning with respect to the formation of partially disentangled features in perception, as well as through the selecting and sculpting of particular policies for enaction (e.g., dopamine as precision weighting, or Kalman gain) (Parr and Friston, 2018a). To the degree diffuse neuromodulator systems both influence and are influenced by overall levels of message passing, these chemicals could be used to adaptively optimize generative models with context sensitivity. Such alterations of cognition and consciousness may be especially powerful with respect to the kinds of serotonergic signaling involved with psychedelic compounds, which is an area of active investigation for further developing IWMT (Safron, 2020c; Safron and Sheikhbahaee, 2021).

### The conscious turbo-code

Turbo-codes are used for reliably sending data over noisy channels (Berrou et al., 1993; Berrou and Glavieux, 1996), with efficiency approaching the Shannon limit, suggesting near optimality. These codes were independently discovered by the cryptography community and Pearl (1982) as methods for approximate Bayesian inference via loopy belief propagation (McEliece et al., 1998). This method of extracting information from noisy signals has found a wide range of uses, including with respect to wireless communication standards. Perhaps these codes were also discovered by natural selection?

Integrated world modeling theory proposes that turbo-coding may be implemented by reciprocal effective connectivity between auto-associated cortical hierarchies, entailing shared reduced-dimensionality latent feature spaces among coupled autoencoders (Supplementary Appendix Figure B). Mechanistically, this would be realized by the formation of large-scale synchronous complexes as self-organizing harmonic modes (SOHMs) over connectivity backbones, some of which may entail action-oriented body maps (i.e., lateral parietal cortices) and visuospatial modeling (i.e., posterior medial cortices). Algorithmically, this would correspond to the calculation of approximate joint posteriors—and maximally likely (MAP) estimates derived thereof—via loopy belief propagation. Functionally, this would correspond to a series of estimated world states of sufficient reliability to form bases for action selection (Vul et al., 2014). Experientially, this would correspond to the stream of consciousness. (Note: While all synchronous complexes could potentially be interpreted as engaging in turbo-coding on some level of abstraction, IWMT suggests that only turbo-codes spanning multiple modalities are likely to be capable of generating conscious experiences).

The high-bandwidth message passing required for conscious turbo-coding may be enabled by the brain’s rich-club, which consumes up to 50% of cortical metabolism (Heuvel et al., 2012). Theoretically, this metabolic expense may be (evolutionarily) justified by reducing the overall number of (noisy) neuronal signal transactions required to achieve adequately reliable perceptual inference, so increasing overall efficiency, and perhaps more importantly, decreasing latencies with respect to action selection. Perhaps even more importantly, turbo-coding over frontal-parietal networks may enable the inferential synergy required for consciously accessible experiences, and potentially the imagination of counterfactual scenarios (Buchsbaum et al., 2012; Schmidhuber, 2012; Pearl and Mackenzie, 2018), so facilitating (a) causal reasoning, (b) planning, and (c) ‘offline’ learning (e.g., self-supervised training via imaginative self-play).

Different rhythmic frequency bands may entail different kinds of information with respect to conscious turbo-codes. When beta complexes are cross-frequency phase coupled within alpha rhythms in posterior cortices, this may correspond to cross-modal message passing across the entire sensorium of the organism, organized within egocentric spatial reference frames, entailing consciousness (i.e., an experienced world) (Figure 2). When these alpha and beta complexes are further orchestrated by theta rhythms from the hippocampal/entorhinal system and its “big loop recurrence” with frontal cortices (Koster et al., 2018), this may correspond to action-driven perception (including simulated actions), and reflective access via comparisons amongst conscious states (Safron, 2021b; Safron et al., 2021a).

Thus, turbo-coding may help to explain the functional significances of some of the mechanisms enabling consciousness. However, these modeling efforts may themselves have a further (circular) causal significance in that they may help to facilitate the conditions that enable them. Under normal circumstances, only coherent and well-evidenced world models are likely to enable loopy message passing to efficiently converge upon (approximate) posteriors, which in turn allow consciously experienced world models to arise. Perhaps similarly to the development of mutually related capacities for spatiotemporally and causally coherent world modeling, this kind of circular bootstrapping suggests that inferential and learning capacities may increase non-linearly, potentially resulting in relatively abrupt (or punctuated) phase transitions for the evolution of consciousness (Isler et al., 2018).

In this view, consciousness emerges from an auto-associative network of coupled generative decoders, connected together to constitute a turbo-code. When message passing is forced to converge via synchrony—and where synchrony emerges from convergent message passing—this may entail maximal *a posteriori* estimates as coherent/discrete vectors with maximal control in governing overall system evolution, sampled from probabilistic spatial-temporal-causal world models. Thus, consciousness (as turbo-code) may not only govern perception as Bayesian model selection, but also action selection (broadly construed to include thought as covert ‘behavior’).

## Future directions for integrated information theory and global neuronal workspace theory?

Integrated world modeling theory proposes that FEP-AI can be used as a framework for synergistically combining leading theories of consciousness, specifically focusing on IIT and GNWT. Below we will discuss some of the ways in which our understandings of the physical and computational bases of consciousness may be advanced through this synthesis, and then move on to discuss how these principles may also lead to potential advances in artificial intelligence.

### Modules and workspaces as complexes of integrated information; potential physical substrates of consciousness

Global neuronal workspace theory describes how global workspaces allow otherwise isolated specialist modules to exchange information. However, the dynamics by which local modules and global workspaces interact remain poorly understood. IIT describes how complexes of effective connectivity can have varying degrees of cause-effect power upon themselves. (For further details, please see Supplementary Appendix, “A review of IIT terminology”). However, the functional relationships between complexes of integrated information remain poorly understood. With FEP-AI as an integrative framework, it may be possible to combine GNWT’s emphasis on function and IIT’s emphasis on dynamics in mutually informative ways. A potentially promising avenue is to apply IIT’s analytic approaches to modules and workspaces as complexes with varying degrees of irreducible self-cause-effect power, including with respect to the ways integrated information varies over the course of cognitive cycles. (For further details, please see Supplementary Appendix, “Evaluating GNWT’s local modules and global workspaces in terms of the axioms of IIT”).

Both local modules and global workspaces can be viewed as constituting complexes of integrated information with varying amounts of irreducible self-cause-effect power (phi). The extent to which modules have more or less phi would specifically depend on the phase of cognitive cycles. Specifically, if “ignition” events correspond to the breakdown of local modularity via the formation of larger complexes of effective connectivity, then we would expect the relative phi for local modules and global workspaces to vary in an inverse fashion. IIT might view this changing modularity as trading off consciousness level between modules and workspaces, with separate modules entailing consciousness when they represent phi maxima, but with these consciousnesses being replaced with a single consciousness when workspace dynamics are present. IWMT and GNWT, in contrast, would only view large-scale workspaces as being capable of supporting conscious experiences.

Integrated information theory, in contrast to GNWT, does not view consciousness as corresponding to a global workspace, but only a posterior “hot zone” as constituting a phi maximum (Boly et al., 2017). The involvement of frontal cortices may be important for instantiating workspace dynamics of a more global nature in terms of widespread availability of information, but according to IIT, these systems would not themselves represent physical substrates of consciousness. IWMT agrees with IIT that basic phenomenality likely centers on posterior cortices, and also agrees with GNWT that frontal cortices are likely crucial for enabling conscious access and autonoetic awareness.

However, IWMT disagrees with IIT that a given module would necessarily be conscious if it constitutes a complex that maximize integrated information (Phi). Rather, modules may be conscious only if they entail integrated models with spatial, temporal, and causal coherence for embodied systems and their relationships to environments in which they are embedded. Given the previously discussed properties of posterior medial cortices, synchronous activity within posterior hot zones could represent an instance of a (large) module being conscious when not participating in global workspace dynamics via the frontal lobes. However, this could also be viewed as a primarily semantic argument, as complexes capable of synergistically integrating information across occipital, temporal, and parietal cortices could reasonably be said to be functioning as ‘global’ workspaces. Perhaps some disputes between GNWT and IIT may be partially resolved by attempting to be more precise about how widespread integration must be to ‘count’ as global.

### Cognitive cycles and fluctuating substrates of consciousness?

In mammals, “posterior hot zones” (Boly et al., 2017) may be both necessary and sufficient for generating consciousness (as integrated world modeling process), and these (both competitive and cooperative) attractor-formation processes may tend to be strictly dominated by dynamics within posterior association cortices. However, by coupling with posterior areas, frontal cortices could help influence the specific compositions of maximal complexes on their timescales of formation. Frontal cortices may be able to influence posterior attracting networks before maximal coherence/integration is achieved, so defining spatial and temporal grains for qualia generation, enabling intentional control of attention, working memory, and action selection. When this effective coupling involves driving of frontal cortices by posterior complexes, this information may also be made more globally available for the sake of higher-order modeling. In these ways, IWMT is also in agreement with GNWT regarding the importance of frontal network hubs, although this may be the case for conscious access, rather than the more posterior-located processes that may be responsible for generating coherent streams of experience.

These hypotheses could potentially be tested via transcranial magnetic stimulation applied at different phases of cognitive cycles (Madl et al., 2011; Sasai et al., 2016) in which (possibly theta-coupled) alpha rhythms may alternate across frontal and posterior cortices, assessing whether intervention influences different kinds of either implicit [e.g., via perturbation complexity index (PCI) methods] or explicit modeling (Schartner et al., 2017). Alternatively, evoked complexity could be time-stamped to endogenous potentials as a measure of different kinds of integrative complexity. While PCI measures can potentially be explained without appealing to IIT, they can nonetheless be used as proxies for integrated information. If GNWT and IIT are compatible in the ways suggested by IWMT, then PCI should be higher during periods where workspace dynamics are present. This could potentially be tested by timing the TMS pulse to coincide with ignition events during which large scale integration occurs, or evaluating Lempel-Ziv complexity after putative ignition events such as the p300 (Mashour et al., 2020; Riggins and Scott, 2020). If integrative complexity measures were not found to be higher accompanying workspace dynamics, this could potentially falsify IWMT.

Perhaps relatedly, an unresolved issue within IWMT is whether consciousness (as experience) corresponds to a series of discrete “snapshots” (Crick and Koch, 2003; Madl et al., 2011; Herzog et al., 2016), like a flipbook or sequential frames in a cartoon/comic (Ha and Schmidhuber, 2018). Alternatively, such discretization could reflect a process of consciously accessing—or sampling from, as in inference via Markov chain Monte Carlo (Gershman, 2019; Dohmatob et al., 2020)—an otherwise continuous stream of experience. IWMT’s account of synchronous complexes as entailing turbo-coding between coupled autoencoders suggests that consciousness could either be understood as flows of inference via traveling waves on a fine-grained level, or as self-organizing harmonic modes (SOHMs) when coarse-grained according to the scales at which various forms of functional closure are achieved (Joslyn, 2000; Chang et al., 2019), including those which would allow for the kinds of higher-order cognition involved in conscious access, self-awareness, forms of meta-awareness, acting with awareness, and planning. In terms of the machine learning models described above, ignition events could potentially be viewed as semi-stochastic sampling from latent spaces, used by variational auto-encoders to parameterize generative models in creating novel combinations of features. If these samples are biased according to histories of reward learning, then these events/samples could correspond to neural dynamics (including those entailing consciousness) being driven in directions that are most likely to realize organismic value, given the data of experience. In this way, it could be the case that ignition events themselves generate consciousness as a series of “snapshots,” or maximal *a posteriori* (MAP) estimates from nervous systems viewed as generative models. Alternatively, it could be the case that ignition events correspond to a source of vectors that parameterize generative models that evolve through more continuous updating.

The seemingly continuous nature of the stream of experience could be illusory, actually corresponding to a series of MAP estimates realized by the turbo coding of ignition events, corresponding to a parameterization of sampling operations, with cortical hierarchies functionally understood as coupled variational autoencoders. Or, iteratively forming these largescale attracting-states may instead be a highly efficient (and potentially optimal) means of realizing globally coherent/integrated inference, where organizing behavior based on a series of estimates has been demonstrated to also be highly efficient from a decision-theoretic perspective (Vul et al., 2014). All these perspectives may be accurate, except with respect to different aspects of experience unfolding on different scales. While frontal-mediated conscious access may be discrete, posterior-generated basic phenomenal consciousness may truly be more like a continuous stream of entangled inferences, whose—potentially shockingly limited (Chater, 2018)—richness overflows awareness.

Integrated world modeling theory currently does not have a definitive prediction as to whether the prefrontal cortices (PFCs) ever represent a physical substrate for consciousness as suggested by GNWT. While a “posterior hot zone” may provide both necessary and sufficient conditions for generating experience as suggested by IIT, it is unclear that frontal cortices ought to be considered as separate from these generative processes, particularly during ignition events in which large-scale frontoparietal complexes are observed. Alternatively, it may be the case that frontal cortices are incapable of significantly driving the heavily entangled internal dynamics of posterior cortices on the timescales at which integration occurs, where posterior-centered inter-relations may have enough causal density to establish functional closure with respect to the processes generating coherent (and so experienceable) world models. Considerations of developmental necessity may also be relevant to debates between IIT and GNWT regarding the neural substrates of consciousness. Frontal cortices may potentially be necessary for the initial development of basic phenomenal consciousness, but not for its continued realization after sufficient experience. That is, frontal cortices may be essential for bootstrapping phenomenal consciousness via the construction of coherent world models, but once developed, these experience-generating capacities—but probably not conscious access, contrary evidence notwithstanding (Bor et al., 2017)—may be preserved even with complete disruption of these initially necessary enabling conditions.

Yet another possibility is that frontal cortices may themselves have enough integrative capacity over requisite sources of information that they represent sufficient substrates of consciousness on their own, potentially offering a source of predictions for what posterior cortices are likely to experience in the future (Knight and Grabowecky, 1995; Ha and Schmidhuber, 2018; Wang et al., 2018). This hypothesis of forward-looking PFCs would be consistent with their roles in action selection and motor control through predicting the sensory consequences of movement (Adams et al., 2013). However, for frontal cortices to generate experience on their own, IWMT would require sufficiency with respect to establishing perspectival reference frames with spatiotemporal and causal coherence. Regardless of whether or not frontal cortices are considered to be directly part of subnetworks generating consciousness, the nature of subjective experience will likely heavily depend on their involvement as emphasized by GNWT and higher order theories (Brown et al., 2019; Shea and Frith, 2019). While (very difficult to test) dissociations may be expected with respect to phenomenal consciousness being possible without conscious access, the qualities of experience will depend on their multi-scale interactions with higher order cognitive processes. For example, the act of introspecting will substantially change the nature of what is (a)perceived (e.g., attention; Sperling phenomena) (Manning et al., 2012).

### Bayesian blur problems and solutions; quasi-quantum consciousness?

While the brain probably does not support the kinds of large-scale coherence required for quantum computation (Schmidhuber, 2000; Tegmark, 2000), it may nonetheless be the case that neuronal dynamics can be viewed as emulating quantum-like computations (e.g., annealing) by classical means (Borders et al., 2019; Coyle et al., 2019; Guillet et al., 2019). Machine learning algorithms play a central role in IWMT, and quantum implementations of autoencoders (e.g., as used in error-correcting codes) may be relevant for making further advances in developing functional analogs for the computational properties of brains. Very speculatively, it may even be the case that dynamic reconfigurations of neuronal microtubules could emulate quantum-like computation in orchestrating signaling (e.g., via transportation rates for neurotransmitter containing vesicles) and memory (via synaptic modifications and synaptogenesis), while not themselves involving sustained quantum coherence (cf. Orch OR theories) (McKemmish et al., 2009).

Indeed, quantum mechanics inspired models could have potential relevance to solving the “Bayesian blur problem” (Clark, 2018). That is, how can a probabilistic model generate seemingly unified experience (cf. the intuition underlying the exclusion axiom from IIT) composed of discrete perceptual experiences, rather than a superposition of possibilities? Functionally speaking, it may be desirable for the brain to provide discrete estimates of—or highly precise distributions over—world states for the sake coherent action selection. However, a “Bayesian blur solution” could also be proposed, in that it may also be desirable to maintain full probability distributions with multiple possibilities kept in play for the sake of adaptation and exploration. In considering workspace dynamics as implementing Bayesian model selection, it may be the case that brains obtain the best of both discrete and probabilistic modeling by “dividing and conquering” across different phases of cognitive cycles, or possibly across brain areas (Gazzaniga, 2018; McGilchrist, 2019). Alternating workspace modes—potentially reflected by the formation/dissolution of mesoscale connectomic modularity (Betzel et al., 2016; Safron et al., 2021b)—could allow periods where multiple competing and cooperating hypotheses can remain in play, followed by winner-take-all dynamics when this information is integrated into larger scale networks and models (Cheung et al., 2019), and then “broadcasted” back to modules as they re-form.

Stanislas Dehaene intriguingly (2014) suggested that the formation of workspaces via ignition events could be understood as a kind of phase change akin to those observed in physical systems. He goes onto propose that a potentially productive analogy could be found in models of wave function collapse in quantum physics, where a superposition of possibilities is reduced to a determinate classical world, which IWMT considers to be a promising avenue for future investigation. It may be similarly productive to explore whether multiple interpretations of quantum mechanics apply to varying degrees as abstract descriptions of varying informational modes within minds, understood in terms of varieties of Bayesian model selection and inferential dynamics. That is, conceptualizations from multiple quantum interpretations (Schmidhuber, 2000; Tegmark, 2014; Carroll, 2016) could potentially apply to different aspects of integrated world modeling. Could entanglement be used to model changes in the precision of probabilistic densities as a function of coupling sub-systems? Could more precise distributions (or estimates derived thereof) due to re-entrant signaling from PFCs be used to implement a kind of Copenhagen-style observer-dependent selection of classical phenomena? Could marginalization via self-organizing synchronous complexes be modeled in a similar manner to spontaneous wave function collapse (and quantum Darwinian interpretations)? Could periods of high modularity/segregation for functional connectomes be productively analogized with branching many worlds? Could relationships between fine-grained neuronal message passing and standing wave descriptions exhibit abstract similarities with Bohmian pilot waves (e.g., chained gamma complexes as quantized prediction errors and solutions)? To be clear, these are all (very) highly speculative analogies for information dynamics, and quantum physical phenomena are likely not directly relevant to the brain’s computational abilities in any meaningful sense, given the hot and crowded nature of biological systems (Tegmark, 2000). Nonetheless, such metaphors/models may potentially afford insights into the nature of neuronal information processing and its connections to different aspects of consciousness.

Regarding “consciousness as collapsing agent” theories (to continue with the analogical extension of quantum mechanics described above): If PFC involvement is important for establishing synchronous coherence in posterior cortices, then this process of dimensionality reduction over dynamics may potentially be likened to wave function collapse by a (potentially unconscious) PFC ‘observer.’ That is, the operation/action of conscious access via PFC re-entry may be required for transforming a continuous sea of probabilities into a discrete stream of experience—as the iterated generation of particular qualia. If the “Bayesian blur” problem is overcome in this manner, then experience may not be solely generated by posterior cortices as described above, potentially favoring GNWT’s suggestion that frontal lobes are part of the physical substrates of consciousness. However, this functionality could potentially be achieved at different stages of cognitive cycles, so excluding PFCs from stages where consciousness is generated (cf. dual phase evolution) (Paperin et al., 2011). Another possibility would involve basic phenomenal consciousness being more diffuse/probabilistic without PFC-involvement, but where conscious access is more particular/discrete. But if this kind of PFC-independent modeling lacks sufficient organization with respect to space, time, and cause, then there may be insufficient coherence to result in the appearance of an experienced world. If this were the case, then it would challenge the distinction between phenomenal consciousness and conscious access, and may potentially support some theories emphasizing higher order cognition (LeDoux and Brown, 2017). The evolving adversarial collaboration between IIT and GNWT theorists may potentially provide evidence that could disambiguate some of these matters.

### Mechanisms for integration and workspace dynamics

Integrated world modeling theory views ignition events in terms of the formation of self-organizing harmonic modes (SOHMs), entailing message passing in nervous systems understood as Bayesian belief networks. In this way, the formation of any meta-stable synchronous complex is viewed as both an ignition event and establishment of a kind of workspace, regardless of whether involvement of frontal lobes and ‘global’ ‘access’ are achieved. In all cases, SOHMs are hypothesized to entail loopy belief propagation and marginalization over effectively connected subnetworks. (For more detail, please see Supplementary Appendix, “Micro-dynamics of SOHM-formation via generalized synchrony”). In the case of small ensembles synchronized at fast gamma frequencies, SOHMs may contribute to the communication of prediction errors up cortical hierarchies (Bastos et al., 2012; Scheeringa and Fries, 2019) via quantized packets of information (as sufficient/summary statistics), so establishing marginal message passing regimes (Parr et al., 2019b). In the case of large ensembles synchronized at beta, alpha, and theta frequencies, SOHMs may allow for large-scale updating of beliefs and sources of integrative predictions from deeper portions of generative models.

In terms of mesoscale and macroscale neuronal dynamics, we might expect large-scale SOHMs to be particularly likely to form in proximity to rich-club hubs of the brain with their high degrees of reciprocal connectivity. These core networks have been found to provide backbones of effective connectivity and robust sources of synchronizing dynamics (Castro et al., 2020). Within these highly interconnected systems, signals may be particularly likely to undergo positive feedback amplification, where this explosive signal transduction may be able to temporarily form synchronous complexes capable of integrating information from across the brain and then propagating (or “broadcasting”) this information to the rest of the network as Bayesian beliefs (or priors in predictive coding).

In terms of generalized synchrony, direction of entraining influence may potentially switch between peripheral and core networks before and after critical ignition events (Safron et al., 2021b). Theoretically, peripheral sensory hierarchies may asymmetrically entrain deeper levels with core connectivity, seeding them with ascending prediction errors, communicated via driving inputs at gamma frequencies. In this way, Bayesian model selection would be driven via a process of differential seeding of core states via competition (and cooperation) amongst neuronal coalitions entailing hypotheses regarding latent causes of sensory observations. These discretely updated core states from deep in the heterarchy could then be used to asymmetrically drive peripheral networks. According to IWMT, these core inferences would be communicated at beta frequencies for specific predictions, alpha frequencies for predictions integrated within egocentric reference frames, and theta frequencies for predictions shaped by particular actions (broadly construed to include mental acts such as attentional fixations; Parr et al., 2019a; Safron, 2021b). Thus, SOHMs and the processes by which they form may function as complexes of integrated information and sources of workspace dynamics, so implementing Bayesian model selection on multiple levels. This multi-level selection—which may also be understood in terms of neural Darwinism and dual-phase evolution (Paperin et al., 2011)—may proceed simultaneously over multiple scales, with both global serial and local parallel integration being implemented by SOHMs of varying spatial (and temporal) extents.

It is worth noting that this proposal does not depend on any given account of predictive processing being accurate. For example, it may be the case that descending modulatory inputs at slower frequencies do not necessarily involve predictive explaining away, but could instead be used to allow sensory observations to ascend with more feedforward driving (Heeger, 2017; George et al., 2020)— which would not be incompatible with an interpretation of attending based on precision weighting (i.e., Kalman gain)—as may be the case with respect to theta-gamma cross-frequency phase coupling (Canolty et al., 2010; Buzsáki and Watson, 2012). It may be the case that slower frequencies could be used to either inhibit or promote the relative contributions of different sensory observations—communicated at faster gamma frequencies—to iterative rounds of Bayesian model selection. This kind of adaptive enhancement of prediction errors may help to reconcile predictive processing with findings that consciousness level and phenomenal binding have been associated with increases in gamma power and inter-electrode gamma coherence (Singer, 2001, 2007), potentially realized by mechanisms involving zero-lag phase synchronization (Gollo et al., 2014). Alternatively, it may merely be the case that more precise predictions tend to be accompanied by increased prediction errors, without observations being specifically enhanced through attentional selection mechanisms. In either case, predictive processing diverges with some more well-known ideas in suggesting that gamma-band activity may not itself generate consciousness, but may instead indirectly modulate belief updating at slower frequencies.

### Beyond integrated information?

Integrated information theory has evolved as a theory over two decades of concerted effort, and further refinements and elaborations of the theory are currently being developed. This ongoing evolution has caused some people to question whether IIT’s postulated mechanisms are truly grounded in axiomatic principles of phenomenology (Bayne, 2018), and whether its methods may contain questionable modeling assumptions. Indeed, many of the most practically useful (and highly face valid) phi estimation techniques rely on previous versions of the theory, such as estimating integrated information based on causal density (Barrett and Seth, 2011; Seth et al., 2011). (For a more detailed discussion, please see Supplementary Appendix: “Toward new methods of estimating integrated information”).

Much skepticism regarding IIT has resulted from demonstrations of high phi values being associated with systems for which there are strong *a priori* reasons to suspect a lack of consciousness, such as the kinds of 2D grids used in expander graphs (Aaronson, 2014). Yet such objections to IIT’s validity can be readily handled by considering integrated information to be necessary, but not sufficient for consciousness without the cybernetic grounding suggested by IWMT. However, the potential modeling capacity of even a single 2D grid should not be underestimated (Wang and Roychowdhury, 2019). With respect to the particular example of the dubious consciousness of expander graphs, it should be noted that such systems have many of the properties which may contribute to the computational power of brains, including small-world connectivity (Takagi, 2018), sparseness (Ahmad and Scheinkman, 2019), and ability to support error-correcting codes (Liu and Poulin, 2019). Theoretically, an arrangement of hierarchically organized expander graphs could be used to implement predictive processing and may be functionally equivalent to the kinds of turbo coding adduced by IWMT. Nonetheless IWMT states that such systems will not be conscious unless their functionality enables coherently integrated world modeling, which may be afforded in mammalian brains by posterior medial cortices (Figure 1) with respect to visual phenomenology and a sense of quasi-Cartesian space (Sutterer et al., 2021).

Others have questioned the merit of emphasizing a single measure for the informational dynamics of complex systems (Mediano et al., 2019). This work has challenged the assumption of pairwise causal interactions in networks, instead focusing on dynamical complexity in terms of the decomposition of integrated information into potentially coexisting modes of informational flows. These novel measures reveal that integration processes can be understood as aggregates of multiple heterogeneous phenomena such as informational storage, copy, transfer, erasure, downward causation, and upward causation. Promisingly, these decomposed measures of integrated information could allow for the creation of novel methods for assessing informational dynamics, which may be superior in some use cases.

Integrated world modeling theory agrees with Mediano et al. (2019) that integrated information is not the only valuable way to look at consciousness or complex systems more generally. Nonetheless, aggregations of heterogeneous phenomena can produce wholes that are greater than the sum of their parts. Mind and life are two such phenomena, and this kind of functional synergy may also apply to informational constructs (including mind and life). If integrated information corresponds to self-model-evidence as described by FEP-AI, then this would be a very special measure of dynamical complexity, potentially indicating the ability of whole systems to be both stable, adaptive, and even autonomous (Albantakis, 2017). Indeed, connections between integrated information and self-organized criticality further suggests that we may be dealing with a measure that applies to all systems capable of not just persisting, but evolving (Arsiwalla and Verschure, 2016; Arsiwalla et al., 2017; Hoffmann and Payton, 2018; Takagi, 2018; Safron et al., 2021b).

### Recurrent networks, universal computation, generalized predictive coding, unfolding, and (potentially conscious) self-world modeling

There may be a kind of generalized predictive coding and implicit intelligence at play across all persisting dynamical systems (Schmidhuber, 2000; Wolfram, 2002; Friston, 2019; Friston et al., 2020; Safron, 2020a; Vanchurin, 2020). However, according to IWMT, consciousness will only be associated with systems capable of coherently modeling themselves and their interactions with the world, likely requiring architectures capable of supporting recurrent processing. This is not to say that recurrence is necessarily required for the functionalities associated with consciousness (Doerig et al., 2019), but recurrent neural networks (RNNs) may be a practical requirement, as supra-astronomical resources may be necessary for unrolling an RNN into a functionally equivalent feedforward neural network (FNN) for a system the size of the human brain across even the 100s of milliseconds over which workspace dynamics unfold. Further, the supposed equivalence of feedforward and feedback processes are only demonstrated when unrolled systems are returned to initial conditions and allowed to evolve under identical circumstances (Marshall et al., 2017). These feedforward “zombie” systems tend to diverge from the functionalities of their recurrent counterparts when intervened upon and will be unable to repair their structure when modified. This lack of robustness and context-sensitivity means that unrolling loses one of the primary advantages of consciousness as dynamic core and temporally extended adaptive (modeling) process, where such (integrated world) models allow organisms to flexibly handle novel situations. Further, while workspace-like processing may be achievable by feedforward systems, largescale neuronal workspaces heavily depend on recurrent dynamics unfolding over multiple scales. Perhaps we could model a single inversion of a generative model corresponding to one quale state, given a sufficiently large computational device (even if this structure might not fit within the observable universe). However, such computations would lack functional closure across moments of experience (Joslyn, 2000; Chang et al., 2019), which would prevent consciousness from being able to evolve as a temporally extended process of iterative Bayesian model selection.

Perhaps more fundamentally, one of the primary functions of workspaces and their realization by dynamic cores of effective connectivity may be the ability to flexibly bind information in different combinations in order to realize functional synergies (Singer, 2001; Baars et al., 2013; Greff et al., 2020; Safron et al., 2021b). While an FNN could theoretically achieve adaptive binding with respect to a single state estimate, this would divorce the integrating processes from its environmental couplings and historicity as an iterative process of generating inferences regarding the contents of experience, comparing these predictions against sense data, and then updating these prior expectations into posterior beliefs as priors for subsequent rounds of predictive modeling. Further, the unfolding argument does not address the issue of how it is that a network may come to be perfectly configured to reflect the temporally extended search process by which recurrent systems come to encode (or resonate with) symmetries/harmonies of the world. Such objections notwithstanding, the issue remains unresolved as to whether an FNN-based generative model could generate experience when inverted.

This issue also speaks to the ontological status of “self-organizing harmonic modes” (SOHMs), which IWMT claims provide a functional bridge between biophysics and phenomenology. Harmonic functions are places where solutions to the Laplacian are 0, indicating no net flux, which could be defined intrinsically with respect to the temporal and spatial scales over which dynamics achieve functional closure in forming self-generating resonant modes (Atasoy et al., 2016). [Note: These autopoietic self-resonating/forming attractors are more commonly referred to as “non-equilibrium steady state distributions” in the FEP literature (Friston, 2019), which are derived using different—but possibly related (Wu and Zhang, 2006)—maths.] However, such recursively self-interacting processes would not evolve in isolation, but would rather be influenced by other proto-system dynamics, coarse-graining themselves and each other as they form renormalization groups in negotiating the course of overall evolution within and without. Are SOHM-like standing wave descriptions ‘real,’ or is everything just a swirling flux of traveling waves? Or, are traveling waves real, or is there ‘really’ just an evolving set of differential equations over a vector field description for the underlying particles? Or are underlying particles real, or are there only the coherent eigenmodes of an underlying topology? Even if such an eliminative reductionism bottoms out with some true atomism, from an outside point of view we could still operate according to a form of subjective realism (Carroll, 2016), in that once we identify phenomena of interest, then maximally efficient/explanatory partitioning into kinds might be identifiable (Hoel et al., 2016; Albantakis et al., 2017; Hoel, 2017). Yet even then, different phenomena will be of differential ‘interest’ to other phenomena in different contexts evolving over different timescales.

While the preceding discussion may seem needlessly abstract, it speaks to the question as to whether we may be begging fundamental questions in trying to identify sufficient physical substrates of consciousness, and also speaks to the boundary problem of which systems can and cannot be considered to entail subjective experience. More concretely, do unrolled SOHMs also entail joint marginals over synchronized subnetworks, some of which IWMT claims to be the computational substrate of consciousness? Based on the inter-translatability of RNNs and FNNs, this question appears to be necessarily answered in the affirmative. However, if the forms of functional closure underlying these synchronization manifolds require temporally extended processes that recursively alter themselves (Rocha, 2000; Rudrauf et al., 2003), then it may be the case that this kind of autopoietic ouroboros cannot be represented via geometries lacking such entanglement. Highly speculatively (and well-beyond the technical expertise of this author), SOHMs might necessarily represent kinds of “time crystals” (Everhardt et al., 2019; Chen et al., 2020; Fruchart et al., 2021) whose symmetry-breaking might provide a principled reason to privilege recurrent systems as physical and computational substrates for consciousness. If this were found to be the case, then we may find yet another reason to describe consciousness as a kind of “strange loop” (Hofstadter, 1979, 2007; Lloyd, 2012), above and beyond the seeming and actual paradoxes involved in explicit self-reference.

This kind of self-entanglement would render SOHMs opaque to external systems lacking the cipher of the self-generative processes realizing those particular topologies (Rocha, 2000). Hence, we may have another way of understanding marginalization/renormalization with respect to inter-SOHM information flows as they exchange messages in the form of sufficient statistics (Parr et al., 2019b), while also maintaining degrees of independent evolution (cf. mean field approximation) over the course of cognitive cycles (Madl et al., 2011). These self-generating entanglements could further speak to interpretations of IIT in which quale states correspond to maximal compressions of experience (Maguire and Maguire, 2010). In evaluating the integrated information of systems according to past and future combinatorics entailed by minimally impactful graph cuts (Tegmark, 2016), we may be describing systems capable of encoding data with maximal efficiency (Maguire et al., 2016), in terms of possessing maximum capacities for information-processing via supporting “differences that make a difference.” A system experiencing maximal alterations in the face of minimal perturbations would have maximal impenetrability when observed from without, yet accompanied by maximal informational sensitivity when viewed from within.

If we think of minds as systems of interacting SOHMs, then this lack of epistemic penetration could potentially be related to notions phenomenal transparency (via opacity) (Metzinger, 2009; Limanowski and Friston, 2018), and perhaps “user interface” theories of consciousness (Hoffman and Prakash, 2014). Intriguingly, maximal compressions have also been used as conceptualizations of the event horizons of black holes, for which corresponding holographic principles have been adduced in terms of internal information being projected onto 2D topologies. With respect to the FEP, it is also notable that singularities and Markov blankets have been interpreted as both points of epistemic boundaries as well as maximal thermal reservoirs (Kirchhoff et al., 2018). Even more speculatively, such holography could even help explain how 3D perception could be derived from 2D sensory arrays, and perhaps also experienced this way in the form of the precuneus acting as a basis for visuospatial awareness and kind of “Cartesian theater” (Dennett, 1992; Haun and Tononi, 2019; Safron, 2021b; Sutterer et al., 2021). As described above, this structure may constitute a kind of GNN, utilizing the locality of recurrent message passing over grid-like representational geometries for generating sufficiently informative projections on timescales proportional to the closure of action-perception cycles (Safron, 2020b). And when coupled with lateral parietal cortices (as upper levels of body map hierarchies), these cortical hubs may theoretically (and potentially exclusively) constitute the physical and computational bases of phenomenal consciousness (Safron, 2021c).

## Conclusion

In attempting to expand on the questions raised by IWMT, opinions will surely vary as to whether we have made substantial progress on contributing to a satisfying solution to the Hard problem of consciousness, or the meta-issue as to whether this is even a real problem (Chalmers, 2018). Several open questions remain, which are currently being explored in the context of models of self-consciousness and agentic control (Safron, 2021b), the hippocampal/entorhinal system as a basis for episodic memory/imagination and high-level cognition (Safron et al., 2021a), cognitive/affective development (Ciaunica et al., 2021; Safron, 2021a), and the computational neurophenomenology of psychedelics (Safron, 2020c; Safron and Sheikhbahaee, 2021).

Directions for future study are numerous and varied, but some particularly promising avenues would likely include focusing on the relationships between consciousness and other closely related constructs such as attention and working memory (Wyart and Tallon-Baudry, 2008; Montemayor and Haladjian, 2015; Haladjian and Montemayor, 2016). That is, different forms of consciousness constitute potentially powerful (and flexible) mechanisms for top-down attentional selection, and bottom-up attentional selection mechanisms help to influence which patterns are likely to enter into fields of consciousness. If neural ensembles are capable of ‘resonating’ with dynamic cores (entailing self-world models) by having compatibly aligned activity, then we may expect deeper processing of these consistent (or consonant) patterns. However, we may also have attentional selection via various kinds of “mental actions” (Sandved-Smith et al., 2021; Ramstead et al., 2022), potentially with qualitatively distinct mechanisms such as theta-gamma cross-frequency phase coupling as mediated by hippocampal and frontal brain systems (Safron et al., 2021a).

It has also been suggested that there may be correspondences between IWMT and higher order theories such as Attention Schema Theory (Graziano, 2019; Safron, 2021b), with workspace-supporting networks of structural (and effective) connectivity potentially being understood as supporting both attention and action-oriented body schemas. If this were found to be the case, then it may have relevance for explaining how biological systems handle the “frame problem” of determining the scope of relevance for any given situation. That is, if consciousness is so deeply embodied that it is inherently structures all percepts via their affordance relations, then enactive minds may handle the frame/relevance problem nearly automatically. Regardless of whether such speculations are supported, investigating relationships between attentional selection and consciousness is of crucial importance, as it may provide one of the strongest means of determining the extent to which intelligence may be facilitated by different forms of conscious processing, potentially revealing the adaptive significance(s) that drove their evolution, and possibly suggesting future directions for developing artificial general intelligence.

Perhaps the Hard problem will only be definitively solved when we can settle when different forms of consciousness first evolved. This is an extremely difficult question, as mental states do not leave fossils, but must be inferred from combining assumptions regarding the functional capacities of different information processing systems and their likely behavioral consequences. A broad range of selective pressures may have contributed to the advent of consciousness and further elaborations in conscious cognition as major transitions in evolution:

1. Cognitive arms races between predators and prey (Godfrey-Smith, 2016), where the evolution of jaws in fish may have been a particularly important milestone (Martik et al., 2019).
2. The transition of aquatic animals to land resulting in increased capacity for long-distance vision approximately 380 million years ago, and so increased adaptive significance for being able to plan ahead (MacIver et al., 2017; Mugan and MacIver, 2019).
3. Selection for precise visualization associated with reaching and grasping of food by prosimians with capable hands (Sagan, 1977).
4. Selection for cognition and visualization abilities to facilitate the coordination required for highly social animals (Tomasello, 2014), and perhaps especially pack-hunting species.
5. Selection for planning when standing afforded increased abilities to see ahead (Russon and Begun, 2007). Further selection for visualization may have occurred due to the challenges associated with bipedal running.
6. Increased selection for precise visualizations with tool-use, including with respect to thrown projectiles during hunting. While such abilities are often considered to be separate from explicit cognition, there is also evidence that counterfactual imaginings are important for guiding implicit learning processes for complex motor sequences (Kilteni et al., 2018; MacKay, 2019).

However, while would all represent situations in which expanding the capacities of conscious processing may have undergone selection, it is unlikely that any of these scenarios adequately addresses the first origins of the evolution of consciousness (as integrated world modeling). For further speculations on this matter, see Supplementary Appendix, “A tentative timeline for the evolution-development of consciousness according to IWMT”.

Ginsburg and Jablonka (2019) have suggested a promising approach based on identifying “evolutionary transition markers,” or adaptations which likely require consciousness for their functioning. Capacities for “unlimited associative learning” are proposed to be the clearest candidate for identifying conscious systems, and are suggested to have arisen around the Cambrian explosion among a wide variety of animals, including arthropods. While consciousness would be very likely to increase the flexibility and cumulative nature of learning processes, IWMT currently does not have a clear position as to whether such processing is necessarily conscious. Indeed, the hippocampal/entorhinal system may be the clearest example of a set of adaptations for flexible learning (Safron et al., 2021a), yet many of these functionalities could potentially be realized unconsciously. In brief, IWMT suggests that consciousness first evolved as a means of generating estimates of likely system-world states, conditioned on a causal world model trained via histories of experience with environmental interactions (including vicarious observations of the actions of others). Such a predictive nexus of integrated information (or “dynamic core”) and workspace could potentially help to realize much of unlimited associative learning, but its initial functionality may have primarily been constituted as a “data fusion” mechanism that structures experience for the sake of more adaptive action selection and credit assignment (Safron, 2020b). That is, it could be highly adaptive to be able to identify particular situations with coherent spatiotemporal organization of features with respect to self and world, with unlimited associative learning potentially constituting a secondary functionality. Future work will explore this issue in greater depth.

Integrated world modeling theory was originally developed based on three observations:

1. A substantial degree of convergence across theories of consciousness, but with differences being emphasized over similarities (cf. adversarial collaborations).
2. A substantial degree of convergence between principles of machine learning and computational models of brain functioning.
3. A surprising lack of consideration for the nature of embodiment in attempting to explain how subjective experience could arise from physical systems.

From this perspective, the most promising way forward for consciousness studies would be for different theorists to more deeply engage with opposing points of view and search for opportunities for synergistic explanations. Further, computational principles from machine learning may not only provide a basis for adjudicating between competing claims, but may provide a powerful algorithmic basis for bridging functional and implementational levels of analysis (Figure 2). This approach of “computational neurophenomenology” involves connecting a multi-level understanding of mind to core aspects of experience (Seth, 2021), for which IWMT and compatible theories suggest that the core explananda are likely the generation of a coherent egocentric perspective with a “lived body” at its center (Rudrauf et al., 2017; Williford et al., 2018). Toward this end, if a sufficiently detailed account of the brain as a kind of hybrid machine learning architecture could be obtained, and if this description was consistent with other models on functional, algorithmic, implementational, and phenomenal levels of analysis, then many might finally consider the Hard problem to be solved. I suggest that such an understanding would provide an invaluable reference point for understanding numerous aspects of minds, providing new means for intervention and control, and perhaps even a basis for the greatest project of all: attempting to create conscious artificial intelligence as potentially world-changing technologies, and possibly as ends in themselves.

## Author contributions

The author confirms being the sole contributor of this work and has approved it for publication.
