# Supplementary material for: Integrated world modeling theory expanded: Implications for the future of consciousness
Source: Front Comput Neurosci. 2022 Nov 24;16:642397. doi: 10.3389/fncom.2022.642397 (PMC9730424; doi:10.3389/fncom.2022.642397)
Supplement: Supplementary file 1 [file Data_Sheet_1.pdf]

Supplementary Appendix for “Integrated world modeling theory expanded: Implications for the future of consciousness”

Contents

Autoencoders 2

Figures from the original publication of IWMT 3

A review of IIT terminology 12

Evaluating GNWT’s local modules and global workspaces in terms of the axioms of IIT 14

Micro-dynamics of SOHM-formation via generalized synchrony 16

Towards new methods of estimating integrated information 18

A tentative timeline for the evolution-development of consciousness according to IWMT 21

Appendix references 23

Autoencoders

Autoencoders use encoder networks to compress higher-dimensionality data into lower-dimensionality feature representations (Kramer, 1991), which are then used by generative decoders to reconstruct likely patterns of higher-dimensional data. These generative models are typically trained by minimizing the reconstruction loss between initial input layers of encoders and output layers of generative decoders. Autoencoders allow richer feature descriptions to be generated from highly compressed or noisy inputs, and can also be used to infer missing data (e.g. filling pixels for occluded sensors).

Variational autoencoders separate the low-dimensionality output of encoders into separate vectors for means and variances, from which sample vectors are derived as inputs to generative decoders, so allowing data to be generated with novel feature combinations (Kingma and Welling, 2014; Doersch, 2016). Training proceeds by simultaneously minimizing both the reconstruction loss (as with non-variational autoencoders), as well as the KL-divergence between the posterior distributions and priors—often a unit Gaussian—so preventing overfitting of observed data and inducing more evenly distributed models of latent feature spaces with more interpretable features (Hanson, 1990). Disentangled variational autoencoders have a precision-weighting term applied to the KL-divergence—equivalent to Kalman-gain in Bayesian filtering—increasing or decreasing the degree to which different (combinations of) feature dimensions are weighted, so allowing more reliable/useful information to be more heavily leveraged during training and inference.

Figures from the original publication of IWMT

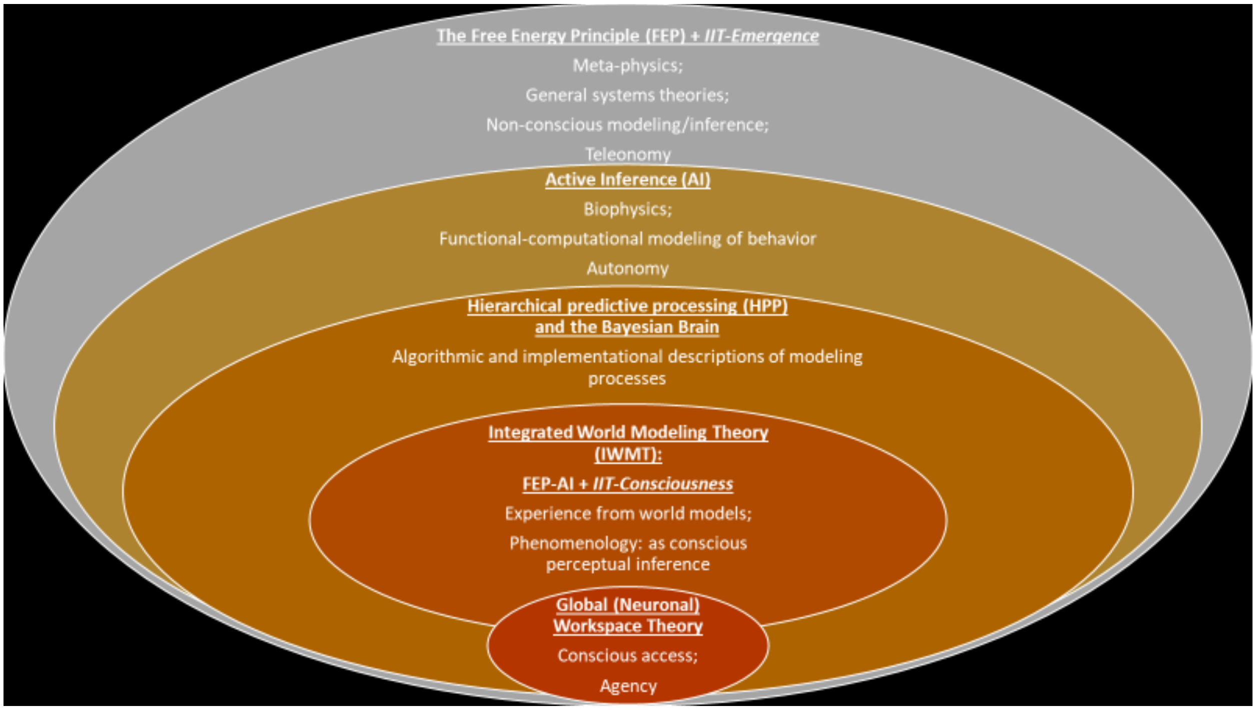

Figure A. Intersections between FEP-AI, IIT, GNWT, and IWMT. (Reprinted with permission from Safron, 2020a.)

The *Free Energy Principle (FEP)* constitutes a general means of analyzing systems based on the preconditions for their continued existence via implicit models. *Integrated Information Theory (IIT)* provides another general systems theory, focused on what it means for a system to exist from an intrinsic perspective. The extremely broad scope of FEP-AI and IIT suggests (and requires for the sake of conceptual consistency) substantial opportunities for their integration as models of systems and their emergent properties. Within the FEP (and potentially within the scope of IIT), a normative functional-computational account of these modeling processes is suggested in *Active Inference (AI)*. *Hierarchical predictive processing (HPP)* provides an algorithmic and implementational description of means by which systems may minimize prediction error (i.e., free energy) via Bayesian model selection in accordance with FEP-AI. Particular (potentially consciousness-entailing) implementations of HPP have been suggested that involve multi-level modeling via the kinds of architectures suggested by *Global Neuronal Workspace Theory (GNWT)*. The concentric circles depicted above are intended to express increasingly specific modeling approaches with increasingly restricted scopes. (Note: These nesting relations ought not be over-interpreted, as it could be argued that HPP does not require accepting the claims of FEP-AI.) This kind of generative synthesis may potentially be facilitated by developing an additional version of IIT, specifically optimized for analyzing systems without concern for their conscious

status, possibly with modified axioms and postulates: *IIT-Consciousness* (i.e., current theory) and *IIT-Emergence* (e.g., alternative formulations that utilize semi-overlapping conceptual-analytic methods). *Integrated World Modeling Theory (IWMT)* distinguishes between phenomenal consciousness (i.e., subjective experience) and conscious access (i.e., higher-order awareness of the contents of consciousness). Non-overlap between the circle containing GNWT and the circle containing IIT-Consciousness is meant to indicate the conceivability of subjectivity-lacking systems that are nonetheless capable of realizing the functional properties of conscious access via workspace architectures. IWMT is agnostic as to whether such systems are actually realizable, either in principle or in practice.

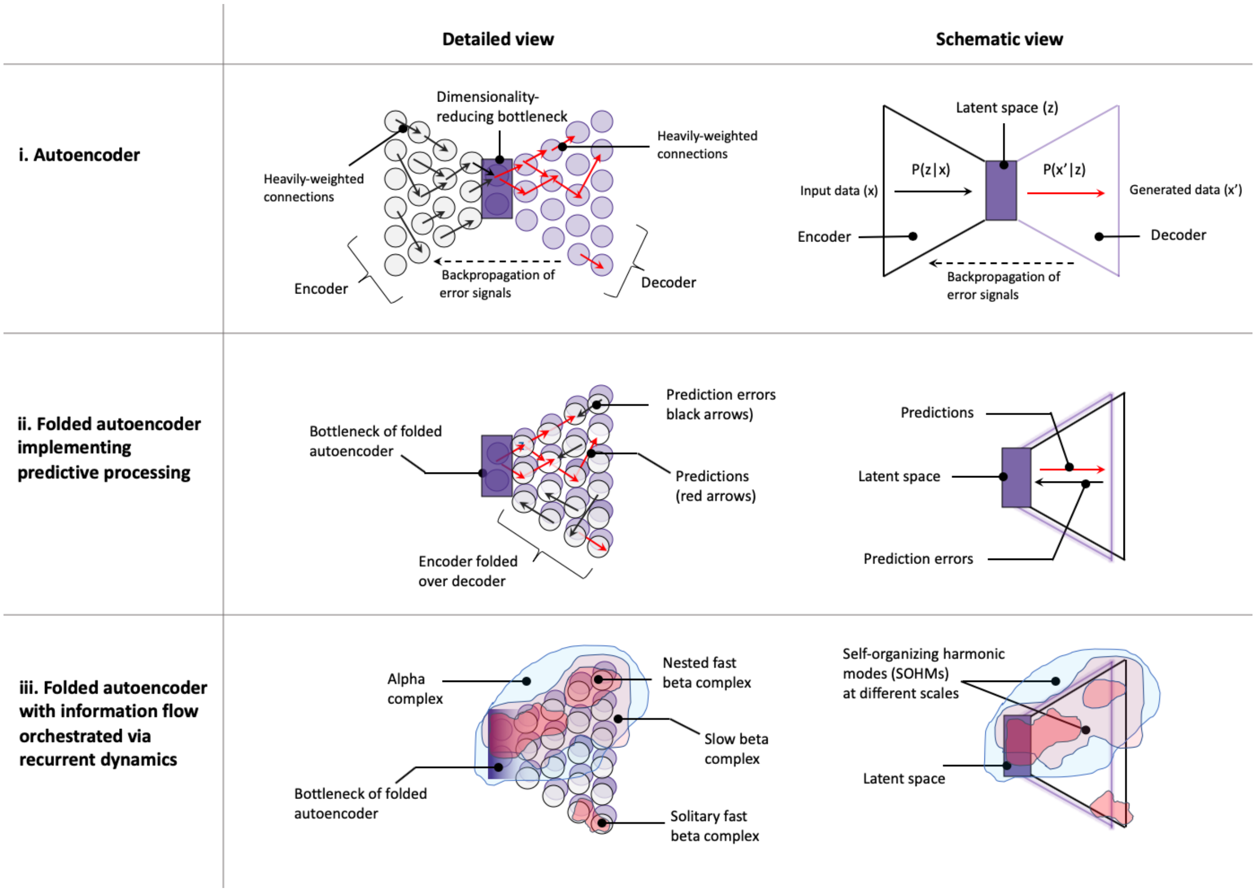

Figure B. Sparse folded variational autoencoders with recurrent dynamics via self-organizing harmonic modes (SOHMs). (Reprinted with permission from Safron, 2020a.)

(i) Autoencoder.

An autoencoder is a type of artificial neural network that learns efficient representations of data, potentially including a capacity for generating more complete data from less complete sources. The encoder compresses input data over stages of hierarchical feature extraction, passes it through a dimensionality-reducing bottleneck and into a decoder. The decoder attempts to generate a representation of the input data from these reduced-dimensionality latent representations. Through backpropagation of error signals, connections contributing to a more inaccurate representation are less heavily weighted. With training, the decoder learns how to generate increasingly high-fidelity data by utilizing the compressed (and potentially interpretable) feature representations encoded in the latent space of the bottleneck portion of the network. In the more detailed view on the left, black arrows on the encoder side represent connections contributing to relatively high marginal likelihoods for particular latent feature space representations, given connection weights and data. Red arrows on the decoder side represent connections with relatively high marginal likelihoods for those reconstructed features, given connection weights and latent space feature hypotheses. While these

autoencoders are fully connected dense networks, particular connections are depicted (and associated probabilities discussed) because of their relevance for predictive processing. Note: Although the language of probability theory is being used here to connect with neurobiologically-inspired implementations, this probabilistic interpretation—and links to brain functioning—is more commonly associated with variational autoencoders, which divide latent spaces into mean and variance distributions parameterized by stochastic sampling operations in generating likely patterns of data, given experience.

(ii) Folded autoencoder implementing predictive processing.

In this implementation of predictive processing, autoencoders are ‘folded' at their low-dimensionality bottlenecks—such that corresponding encoding and decoding layers are aligned—with decoding hierarchies (purple circles) depicted as positioned underneath encoding hierarchies (gray circles). Within a brain, these decoding and encoding hierarchies may correspond to respective populations of deep and superficial pyramidal neurons (Bastos et al., 2012). In the figure, individual nodes represent either units in an artificial network—or groups of units; e.g., capsule networks (Kosiorek et al., 2019)—or neurons (or neuronal groups; e.g., cortical minicolumns) in a brain. Predictions (red arrows) suppress input signals when successfully predicted, and are depicted as traveling downwards from representational bottlenecks (corresponding to latent spaces) along which autoencoding networks are folded. Prediction errors, or observations for a given level (black arrows) continue to travel upwards through encoders unless they are successfully predicted, and so “explained away.” Data observations (i.e., prediction errors) are depicted as being sparser relative to high-weight connections in the (non-folded) encoding network presented above, where sparsity is induced via predictive suppression of ascending signals. This information flow may also be viewed as Bayesian belief propagation or (marginal) message passing (Friston et al., 2017; Parr et al., 2019). In contrast to variational autoencoders in which training proceeds via backpropagation with separable forward and backward passes—where cost functions both minimize reconstruction loss and deviations between posterior latent distributions and priors (usually taking the form of a unit Gaussian)—training is suggested to occur (largely) continuously in predictive processing (via folded autoencoders), similarly to recent proposals of target propagation (Hinton, 2017; Lillicrap et al., 2020). Note: Folded autoencoders could potentially be elaborated to include attention mechanisms, wherein higher-level nodes may increase the information gain on ascending prediction-errors, corresponding to precision-weighting (i.e., inverse variance over implicit Bayesian beliefs) over selected feature representations.

(iii) Folded autoencoder with information flows orchestrated via recurrent dynamics.

This row shows a folded autoencoder model of a cortical hierarchy, wherein neuronal oscillations mediate predictions—potentially orchestrated by deep pyramidal neurons

and thalamic (and striatal) relays—here characterized as self-organizing harmonic modes (SOHMs). This paper introduces SOHMs as mechanisms realizing synchronization manifolds for coupling neural systems (Palacios et al., 2019), and sources of coherent neuronal oscillations and evidence accumulation for predictive processing. Depending on the level of granularity being considered, these predictive oscillations could either be viewed as traveling or standing waves (i.e., harmonics). SOHM-based predictions are shown as beta oscillations forming multiple spatial and temporal scales. These predictive waves may be particularly likely to originate from hierarchically higher levels—corresponding to latent spaces of representational bottlenecks—potentially due to a relatively greater amount of internal reciprocal connectivity, consistent information due to information aggregation, or both. SOHMs may also occur at hierarchically lower levels due to a critical mass of model evidence accumulation allowing for the generation of coherent local predictions, or potentially on account of semi-stochastic synchronization. Faster and smaller beta complexes are depicted as nested within a larger and slower beta complex, all of which are nested within a relatively larger and slower alpha complex. Note: In contrast to standard machine learning implementations, for this proposal of predictive processing via folded autoencoders (and SOHMs), latent space is depicted as having unclear boundaries due to its realization via recurrent dynamics. Further, inverse relationships between the spatial extent and speed of formation for SOHMs are suggested due to the relative difficulties of converging on synchronous dynamics within systems of various sizes; theoretically, this mechanism could allow for hierarchical modeling of events in the world for which smaller dynamics would be expected to change more quickly, and where larger dynamics would be expected to change more slowly.

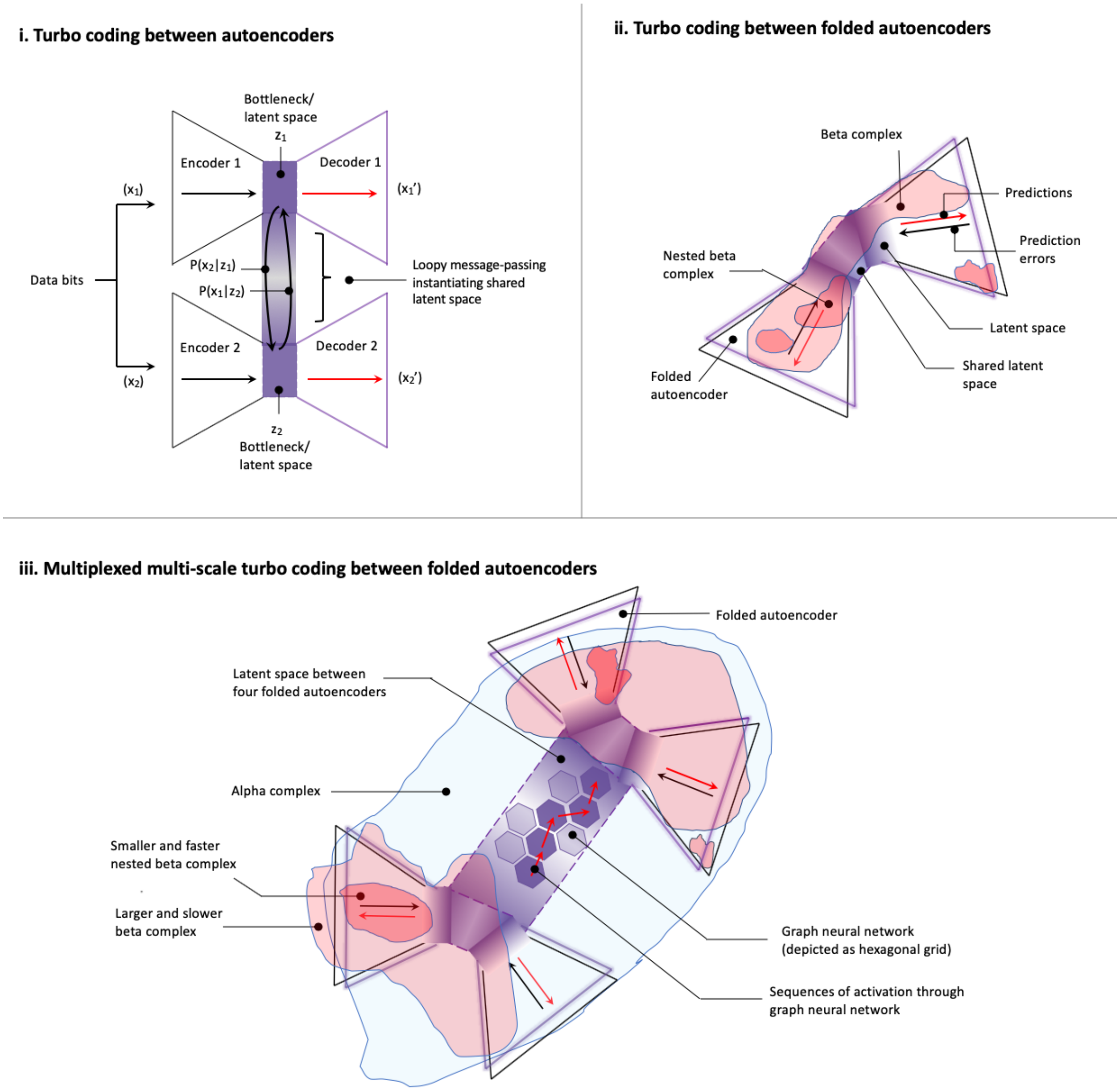

Figure C. Cortical turbo codes. (Reprinted with permission from Safron, 2020a.)
(i) Turbo coding between autoencoders.
Turbo coding allows signals to be transmitted over noisy channels with high fidelity, approaching the theoretical optimum of the Shannon limit. Data bits are distributed across two encoders, which compress signals as they are passed through a dimensionality reducing bottleneck—constituting a noisy channel—and are then passed through decoders to be reconstructed. To represent the original data source from compressed signals, bottlenecks communicate information about their respective (noisy) bits via loopy message passing. Bottleneck z_1 calculates a posterior over its input data, which is now passed to Bottleneck z_2 as a prior for inferring a likely reconstruction (or posterior) over its data. This posterior is then passed back in the other direction

(Bottleneck z_2 to Bottleneck z_1) as a new prior over its input data, which will then be used to infer a new posterior distribution. This iterative Bayesian updating repeats multiple times until bottlenecks converge on stable joint posteriors over their respective (now less noisy) bits. IWMT proposes that this operation corresponds to the formation of synchronous complexes as self-organizing harmonic modes (SOHMs), entailing marginalization over synchronized subnetworks—and/or precision-weighting of effectively connected representations—with some SOHM-formation events corresponding to conscious "ignition" as described in Global Neuronal Workspace Theory (Dehaene, 2014). However, this process is proposed to provide a means of efficiently realizing (discretely updated) multi-modal sensory integration, regardless of whether "global availability" is involved. Theoretically, this setup could allow for greater data efficiency with respect to achieving inferential synergy and minimizing reconstruction loss during training in both biological and artificial systems. In terms of concepts from variational autoencoders, this loopy message passing over bottlenecks is proposed to entail discrete updating and maximal a posteriori (MAP) estimates, which are used to parameterize semi-stochastic sampling operations by decoders, so enabling the iterative generation of likely patterns of data, given past experience (i.e., training) and present context (i.e., recent data preceding turbo coding). Note: In turbo coding as used in industrial applications such as enhanced telecommunications, loopy message passing usually proceeds between interlaced decoder networks; within cortex, turbo coding could potentially occur with multiple (potentially nested) intermediate stages in deep cortical hierarchies.

(ii) Turbo coding between folded autoencoders.

This panel shows turbo coding between two folded autoencoders connected by a shared latent space. Each folded autoencoder sends predictions downwards from its bottleneck (entailing reduced-dimensionality latent spaces), and sends prediction errors upwards from its inputs. These coupled folded autoencoders constitute a turbo code by engaging in loopy message passing, which when realized via coupled representational bottlenecks is depicted as instantiating a shared latent space via high-bandwidth effective connectivity. Latent spaces are depicted as having unclear boundaries—indicated by shaded gradients—due to their semi-stochastic realization via the recurrent dynamics. A synchronous beta complex is depicted as centered on the bottleneck latent space—along which encoding and decoding networks are folded—and spreading into autoencoding hierarchies. In neural systems, this spreading belief propagation (or message-passing) may take the form of traveling waves of predictions, which are here understood as self-organizing harmonic modes (SOHMs) when coarse-grained as standing waves and synchronization manifolds for coupling neural systems. Relatively smaller and faster beta complexes are depicted as nested within—and potentially cross-frequency phase coupled by—this larger and slower beta complex. This kind of nesting may potentially afford multi-scale representational hierarchies of

varying degrees of spatial and temporal granularity for modeling multi-scale world dynamics. An isolated (small and fast) beta complex is depicted as emerging outside of the larger (and slower) beta complex originating from hierarchically higher subnetworks (hosting shared latent space). All SOHMs may be understood as instances of turbo coding, parameterizing generative hierarchies via marginal maximum a posteriori (MAP) estimates from the subnetworks within their scope. However, unless these smaller SOHMs are functionally nested within larger SOHMs, they will be limited in their ability to both inform and be informed by larger zones of integration (as probabilistic inference).

(iii) Multiplexed multi-scale turbo coding between folded autoencoders.

This panel shows turbo coding between four folded autoencoders. These folded autoencoders are depicted as engaging in turbo coding via loopy message passing, instantiated by self-organizing harmonic modes (SOHMs) (as beta complexes, in pink), so forming shared latent spaces. Turbo coding is further depicted as taking place between all four folded autoencoders (via an alpha complex, in blue), so instantiating further (hierarchical) turbo coding and thereby a larger shared latent space, so enabling predictive modeling of causes that achieve coherence via larger (and more slowly forming) modes of informational integration. This shared latent space is illustrated as containing an embedded graph neural network (GNN) (Liu et al., 2019; Steppa and Holch, 2019), depicted as a hexagonal grid, as a means of integrating information via structured representations, where resulting predictions can then be propagated downward to individual folded autoencoders. Variable shading within the hexagonal grid-space of the GNN is meant to indicate degrees of recurrent activity—potentially implementing further turbo coding—and red arrows over this grid are meant to indicate sequences of activation, and potentially representations of trajectories through feature spaces. These graph-grid structured representational spaces may also afford reference frames at various levels of abstraction; e.g., space proper, degrees of locality with respect to semantic distance, abductive connections between symbols, causal relations, etc. If these (alpha- and beta-synchronized) structured representational dynamics and associated predictions afford world models with spatial, temporal, and causal coherence, these processes may entail phenomenal consciousness. Even larger integrative SOHMs may tend to center on long-distance white matter bundles establishing a core subnetwork of neuronal hubs with rich-club connectivity (Heuvel and Sporns, 2011). If hippocampal-parietal synchronization is established (typically at theta frequencies), then bidirectional pointers between neocortex and the entorhinal system may allow decoders to generate likely patterns of data according to trajectories of the overall system through space and time, potentially enabling episodic memory and imagination. If frontal-parietal synchronization is established (potentially involving theta-, alpha-, and beta- synchrony), these larger SOHMs may also correspond to

“ignition” events as normally understood in Global Neuronal Workspace Theory, potentially entailing access consciousness and volitional control.

A review of IIT terminology

The IIT formalism begins by choosing a candidate system—as substrate for consciousness or maximal complex with irreducible cause-effect power over itself—and then identifying the intrinsic cause-effect structure of that (proto-)system as a set of elements. The cause-effect structure for (and definition of) a system is composed of all maximally irreducible cause-effect distinctions (i.e., "concepts", or "MICE" repertoires), which in recent developments within IIT have been extended to include relations between concepts/distinctions (Haun and Tononi, 2019). In this way, each and every intrinsically existing system is defined by a single (maximally irreducible) cause-effect structure, composed of at least one and possibly many distinctions/concepts/MICEs. The cause-effect structure and corresponding Phi value can be computed for any candidate system, but only candidates that maximize integrated information (as Phi, or self-cause-effect power) exclusively qualify as intrinsically existing systems. These maximal complexes (of experience) are referred to as MICS in IIT 3.0, and are hypothesized to correspond to the physical substrates of consciousness.

In this way, the interrelationships between the conceptual entities of IIT for analyzing potentially conscious systems may be summarized as follows:

1. *Elements*: Physical constituents of a system that can be manipulated and observed, which may or may not contribute to a system's cause-effect structure.
2. *Mechanisms*: Individual elements or sets of elements that have irreducible cause-effect power by virtue of constraining both past and future states of other elements within a candidate system.
3. *Cause-effect repertoire*: Probability distributions over both past and future states for a mechanism, given its present state.
4. *Concepts/distinctions*: Mechanisms and their associated phi values and the intrinsic information (as distinctions) specified over their purviews with respect to repertoires of causes and effects within a candidate system, understood as an ability to maximally (irreducibly) constrain past and future states, given present states (i.e., MICE, or maximally irreducible cause effect repertoires).
5. *Cause-effect structure*: The set of all concepts/distinctions for all mechanisms within a system.
6. *Conceptual structure*: All cause-effect repertoires within a candidate system (which may potentially be reducible to simpler systems), so including all the intrinsic (cause-effect) information from the mechanisms of which a system is composed.
7. *Complex*: Candidate system with maximal Phi across all possible system definitions, existing for itself intrinsically, corresponding to the physical substrate of consciousness.

8. *MICS*: The maximally irreducible cause-effect structure entailed by a complex, corresponding to "what it is like" to be that intrinsically existing set of mechanisms, composed of *MICE* repertoires, which correspond to the individual phenomenal distinctions within experience.

In this way, IIT begins from axioms regarding the nature of consciousness, postulates corresponding mechanisms with those properties, and then formally analyzes candidate systems from a system-internal perspective in order to identify sets of mechanisms with maximal claim to intrinsic existence as nexuses of self-cause-effect-power (i.e., integrated information). A maximal complex is suggested to constitute a physical substrate of consciousness, entailing a MICS as the totality of experience (as concept) unfolding at a particular temporal and spatial grain, within which particular qualitative distinctions can be identified (i.e., MICE repertoires).

IWMT supports this process for identifying maximally explanatory systems and even physical substrates of consciousness, except a MICS would entail subjectivity if (and only if) it corresponded to a joint probability distribution (or maximal estimate derived thereof) from a generative model with spatial, temporal, and causal coherence for system and world. In this Bayesian/FEP-AI interpretation of IIT, MICE repertoires would correspond to particular factorizations of generative models, which would have phenomenal content by virtue of reflecting sensorimotor states for embodied-embedded agents. That is, the reason there may be "something that it is like" to be such a generative model, is because neural dynamics are coupled to (or entrained with) sensors and effectors (which are in turn coupled to system-world dynamics), so providing means of modeling spatiotemporally (and causally) coherent patterns in system and world. In this way, spatiotemporal (and causal) coherence for both system and world allows for the possibility of phenomenal consciousness through the alignment/representation of (or generalized synchrony between) those coherences.

Evaluating GNWT's local modules and global workspaces in terms of the axioms of IIT

1. Intrinsic existence
    a. Modules have cause-effect power upon themselves; modules depend upon workspaces in order to have cause-effect power upon each other.
    b. Workspaces have cause-effect power upon themselves and the modules with which they couple. That is, workspaces emerge via processes of self-organization involving reentrant signaling (Edelman et al., 2011).
2. Composition
    a. Modules have internal structure by which they exert cause-effect power on themselves, as well as other modules via workspaces.
    b. Workspaces have internal structures by which they exert cause-effect power upon themselves, the compositions of which depend on the modules with which they couple. That is, workspaces have particular compositions, so allowing them to possess information about other structured phenomena, such as the compositions of the world (Whyte and Smith, 2020).
3. Information
    a. Modules have particular cause-effect structures that differentiate their specific compositions from other possible configurations; modules depend on workspaces to share this intrinsic information with each other.
    b. Workspaces have particular cause-effect structures that specify particular large-scale state compositions, which inform and are informed by the modules with which they couple in the context of cognitive cycles of perception and action selection, so acting as large-scale systemic causes (Madl et al., 2011).
4. Integration
    a. Modules specify unified cause-effect structures that are irreducible to sub-components.
    b. Workspaces specify unified cause-effect structures that are irreducible to sub-components, including information from the modules with which they couple. That is, workspaces are wholes that are greater than the sum of their parts (Chang et al., 2019).
5. Exclusion
    a. Modules specify particular cause-effect structures whose degree of intrinsic irreducibility evolves over particular spatial and temporal grains, depending on their ability to couple with each other and workspaces.

b. Workspaces specify particular cause-effect structures whose degree of intrinsic irreducibility evolves over particular spatial and temporal scales, with particular community structures depending on both internal and external dynamics (Betzel et al., 2016).

Micro-dynamics of SOHM-formation via generalized synchrony

Considering their potential central role for driving neural evolution, it is worth considering in detail the dynamics by which synchronous complexes form. Some recent promising work in this direction can be found in models of synchronous dynamics emerging through collaborative inference among interacting oscillators (Palacios et al., 2019). This kind of coordination in the Free Energy Principle and Active Inference (FEP-AI) framework is often described in terms of the near ubiquitous phenomenon whereby systems minimize free energy through generalized synchrony (Strogatz, 2012; Kachman et al., 2017), as first demonstrated by Huygens with respect to pendulum clocks (Oliveira and Melo, 2015; Willms et al.). Here, I will provide an informal sketch of how SOHM-formation might proceed and the potential functional consequences that may result from these processes:

1. Let us consider a set of neuronal oscillators that are initially maximally desynchronized, but which gradually acquire a shared absorbing rhythm.
2. If neuronal oscillators happen to interact while phase-aligned, then they will be more likely to be able to drive activity due to stimulation happening within windows wherein temporal summation is possible.
3. If reciprocal connectivity is present between phase-aligned neuronal oscillators, then there is a potential for positive feedback and self-sustaining rhythmic activity.
4. In this way, initial locally synchronized ensembles may be able to spread synchronous organization as the stability of their rhythmic activity provides opportunities for additional phase-aligned oscillators to become entrained to the absorbing rhythm.
5. However, this potential for positive feedback must be accompanied by negative feedback mechanisms (e.g. GABAergic interneurons) to maintain adaptive exploration of state spaces (Friston et al., 2012), and also to avoid the kinds of explosive percolation events observed in clinical conditions like epilepsy (Bartolomei and Naccache, 2011; D'Souza and Nagler, 2015; Safron, 2016; Kinouchi et al., 2019).
6. During periods of minimal synchronization, we may expect synchronizing ensembles to be maximally sensitive to signals at any phase (Lahav et al., 2018), but with minimal abilities to drive coupling systems.
7. During periods of maximum synchronization, we may expect synchronizing ensembles to be maximally sensitive to phase-aligned signals—as well as minimally sensitive to non-phase-aligned signals—and with maximal abilities to drive coupling systems.

8. During intermediate periods where synchronization dynamics are accumulating, we may expect sensitivity to a greater diversity of signals, with a potential capacity for mutual influence between coupling systems during this bifurcation window.

With respect to belief propagation in Bayesian networks, all of this could potentially be understood as a means of enabling and constraining belief propagation, influencing which messages will be likely to be exchanged on what timescales.

Towards new methods of estimating integrated information

According to IWMT, IIT's maximal complexes and GNWT's workspaces are emergent eigenmodes of effective connectivity (Friston et al., 2014; Atasoy et al., 2018), or self-organizing harmonic modes (SOHMs) (Safron, 2020). Considering the network properties of brains (Heuvel and Sporns, 2011), these SOHMs are likely to be centered around deep portions of hierarchical generative models which may enable (via turbo-coding) convergence upon approximate posteriors (and empirical priors for subsequent rounds of Bayesian model selection). If this integrative view is accurate, then it may provide new means of evaluating phi estimation methods (Tegmark, 2016), with significance for both basic research and clinical practice. Reliable means of estimating phi are necessary for practical applications, as the formally-prescribed calculations are NP-hard (Mayner et al., 2018; Toker and Sommer, 2019), and so require simplifying assumptions for even modestly-sized causal networks. Nature, in contrast may implicitly perform such calculations nearly 'for free' via Hamilton's principle of least action. Unfortunately, Seth and colleagues (2019) have found radically different integrated information estimates can be derived with seemingly reasonable modeling assumptions. If IWMT's proposal is correct—that phi corresponds to self-model-evidence; a claim which has recently received endorsement from the primary architect of FEP-AI (2020)—then applying different integration estimation methods to Bayesian networks may provide a ground truth for adjudicating between different estimation approaches.

If IWMT's proposal is correct in suggesting a complex with maximally irreducible cause-effect power (i.e., a MICS) is also a maximally informative subgraph, then this correspondence could provide further means of estimating integrated information (phi). [Note: IWMT does not necessarily ascribe to the particular definition of phi provided by IIT 3.0, as a case could be made for meaningful informational synergy being better reflected in other ways in different circumstances.] According to FEP-AI, neural dynamics can be viewed as implementing approximate Bayesian inference, where activation cascades constitute a message passing regime (Friston et al., 2017; Parr et al., 2019). Theoretically, differential rates of message passing may automatically discover maximally connected subnetworks (Mišić et al., 2015), and in doing so, converge on processes of variable elimination (Koller and Friedman, 2009). In variable elimination, marginal information from factors are progressively integrated into an induced graph—or maximal clique—thereby providing a maximally likely a posterior (MAP) estimate from the overall belief network. If maximal complexes tend to be centered on these maximal cliques (or are equivalent to them) these internally-directed cause-effect structures could have actual semantic content by virtue of

containing (or entailing) MAP estimates over hidden causes that define self and world for embodied-embedded agents.

It is unclear whether these specific correspondences between probabilistic graphical modeling techniques and concepts from IIT will be found to be valid. However, if IWMT is accurate in claiming that self-cause-effect power in IIT corresponds with self-model-evidence in FEP-AI, then relative merits for different phi estimation techniques could be evaluated based on their abilities to track the inferential properties of Bayesian networks. Specifically, metrics of quality for phi estimates could be indicated by quicker convergence times for loopy message passing, more precise posterior distributions and accurate MAP estimates, and enhanced learning rate or inferential power more generally (Koller and Friedman, 2009).

IWMT's synthesis could also potentially lead to novel phi estimation methods based on modeling processes by which complexes of integrated information emerge via self-organization (i.e., SOHMs). Although a detailed handling is beyond the scope of the present discussion, useful means of estimating phi and modeling SOHM/complex formation may potentially be found in flow networks (cf. max-flow-min-cut theorem) (Dantzig and Fulkerson, 1955; Garg et al., 1996; Hoffman, 2003) and other kinds of physical systems. Further, game theoretically informed constructs such as Shapley centrality have been used in the study of dynamic networks (Chen and Teng, 2017; Ghorbani and Zou, 2020), and such measures may be relevant for modeling processes by which complexes of cause-effect power emerge. Estimation techniques inspired by these kinds of analogies may potentially be more computationally tractable than other phi estimation methods, and may also provide further bridges between IIT and FEP-AI (and thereby GNWT when workspace dynamics are considered to represent Bayesian model selection).

Speculatively, it appears that there may be potentially fruitful correspondences between maximal complexes in IIT and the identification of the kernel (and/or nucleolus) of the core of a game (Schmeidler, 1969; Maschler et al., 1979; Maschler, 1992). But with respect to the core of a cooperative game, integrated information as cause-effect power would refer to a nexus of bargaining influence amongst players in a coalition. Below, I provide an informal sketch of how such an analysis might proceed:

1. Persisting neuronal dynamics may be modeled as quasi-agents, with patterns constituted by implicit models for their continued existence (i.e., preserving their Markov blankets).
2. For these quasi-agents, utility would be defined in terms of generating self-model-evidence, and cost would be defined in terms of prediction error—within a generalized Darwinian framework, these implicit utility functions would also be fitness functions (Safron, 2019a).

3. Hamilton's principle of least action implies that each dynamical pattern will always choose its best response for minimizing free energy (Kaila and Annila, 2008; Friston, 2019).
4. In this self-prediction game, stable coalitions (as cores) would correspond to Nash equilibrium solutions, wherein competing and cooperating neuronal quasi-agents are not incentivized to leave the grand coalition, because doing so would increase free energy.
5. When stable game theoretic equilibria can be found, the center of gravity for these geometries would also represent Shapley values (Maschler et al., 1979), or solutions that balance the respective utilities and bargaining power for participating quasi-agents.
6. If the game being played entails hypotheses regarding latent causes, and if bargaining power is a function of predictive ability, then this Shapley value may also represent a precision-weighted probabilistic model of world states, or maximal (MAP) estimate derived thereof.

While admittedly speculative and underdeveloped, such a game theoretic derivation of complexes of integrated information—functioning as workspaces—could help provide a further cybernetic-computational grounding for IIT (and GNWT), answering the question of why there may be "something that it is like" to be a maximal complex, or workspace, or any physical system. That is, if the formation processes of workspaces and complexes entail 'calculation' of probabilistic estimates of system-environment states, then for an embodied-embedded agent this could correspond to a "lived" world. Alternatively (and very speculatively), similar analyses (and implications) may potentially be derived by modeling neural dynamics as entailing a kind of prediction market (Conitzer, 2012), with estimated prices representing probabilities for the sufficient statistics for world models. These kinds of handlings of integrative processes may render notions of "neuronal coalitions" (Crick and Koch, 2003) as something more than a mere metaphor, and could also give new meaning to Edelman's (2011) description of consciousness as being realized by a "dynamic core" (Safron, 2019b).

A tentative timeline for the evolution-development of consciousness according to IWMT

- Being a model where dynamics entail implicitly predictive modeling processes (everything that exists; ~13.45 billion years old within this universe); almost certainly nothing that it is like.
- Being a model that has a model in the form of complex inner states that track engagements with the world in an adaptive (i.e., predictive) fashion, but without centralized integration structures (all life; ~3.7 billion years old); probably nothing that it is like.
- Being a model that has a model with centralized integration structures (e.g. nervous systems), but not ones capable of generating coherent world models (all nervous systems; > 1 billion years old); proto-awareness; probably nothing that it is like.
- Being a model that has a model with sub-models that generate sensorimotor states for the organism's embodiment, but is incapable of coherently modeling causal dependencies between system-world relations (all vertebrates; ~560 million years old); proto-creature consciousness; unclear whether or not there is anything that it is like.
- Being a model that has a model that has sub-models capable of producing phenomenal coherence (all animals with well-developed hierarchical memory systems; ~430-200 million years old); basic phenomenality and beginnings of consciousness proper; something that it is like.
- Being a model that has a model that has sub-models capable of modeling phenomenally coherent sub-models (hominids (and possibly some other non-human animals); ~1-2 million years old); higher order consciousness; introspectable "something that it is like", where this accessibility and likeness qualitatively changes the nature of subjectivity.
- Being a nested modeling process capable of generating counterfactually rich causal models with respect to both explicit subjectively experienced and intersubjectively entrained modeling efforts (all humans; ~70,000 years old?); ancestral/child consciousness and the beginning of agency proper; capable of imagining what it might be like under counterfactual possibilities.
- Being a nested modeling process capable of supporting self-processes in an entrainment relation with (sharable) diachronic narratives and explicit recursive self-reference, including with respect to cultural contexts (~10,000-3,000 years old?); modern self/other-consciousness as an evolving generative process capable of creating/constructing an enormous variety of meta-aware intentionally-shaped experiences; many things that it is like and could be like.

- Being an even more elaborate set of intersecting (and sometimes nested) modeling processes (possible future artificial intelligences, and possibly some advanced alien species (if they exist)); unclear what it is/will-be like.

Appendix references

Atasoy, S., Deco, G., Kringelbach, M. L., and Pearson, J. (2018). Harmonic Brain Modes: A Unifying Framework for Linking Space and Time in Brain Dynamics. *Neurosci. Rev. J. Bringing Neurobiol. Neurol. Psychiatry* 24, 277–293. doi: 10.1177/1073858417728032.

Bartolomei, F., and Naccache, L. (2011). The global workspace (GW) theory of consciousness and epilepsy. *Behav. Neurol.* 24, 67–74. doi: 10.3233/BEN-2011-0313.

Bastos, A. M., Usrey, W. M., Adams, R. A., Mangun, G. R., Fries, P., and Friston, K. J. (2012). Canonical microcircuits for predictive coding. *Neuron* 76, 695–711. doi: 10.1016/j.neuron.2012.10.038.

Betzel, R. F., Fukushima, M., He, Y., Zuo, X.-N., and Sporns, O. (2016). Dynamic fluctuations coincide with periods of high and low modularity in resting-state functional brain networks. *NeuroImage* 127, 287–297. doi: 10.1016/j.neuroimage.2015.12.001.

Chang, A. Y. C., Biehl, M., Yu, Y., and Kanai, R. (2019). Information Closure Theory of Consciousness. *ArXiv190913045 Q-Bio*. Available at: http://arxiv.org/abs/1909.13045 [Accessed October 26, 2019].

Chen, W., and Teng, S.-H. (2017). Interplay between Social Influence and Network Centrality: A Comparative Study on Shapley Centrality and Single-Node-Influence Centrality. *ArXiv160203780 Phys.* Available at: http://arxiv.org/abs/1602.03780 [Accessed December 11, 2019].

Conitzer, V. (2012). Prediction Markets, Mechanism Design, and Cooperative Game Theory. *ArXiv12052654 Cs*. Available at: http://arxiv.org/abs/1205.2654 [Accessed January 29, 2020].

Crick, F., and Koch, C. (2003). A framework for consciousness. *Nat. Neurosci.* 6, 119–126. doi: 10.1038/nn0203-119.

Dantzig, G. B., and Fulkerson, D. R. (1955). On the Max Flow Min Cut Theorem of Networks. Available at: https://www.rand.org/pubs/papers/P826.html [Accessed June 13, 2019].

Dehaene, S. (2014). *Consciousness and the Brain: Deciphering How the Brain Codes Our Thoughts*. New York, New York: Viking.

Doersch, C. (2016). Tutorial on Variational Autoencoders. *ArXiv160605908 Cs Stat.* Available at: http://arxiv.org/abs/1606.05908 [Accessed March 27, 2020].

D'Souza, R. M., and Nagler, J. (2015). Anomalous critical and supercritical phenomena in explosive percolation. *Nat. Phys.* 11, 531–538. doi: 10.1038/nphys3378.

Edelman, G., Gally, J. A., and Baars, B. J. (2011). Biology of consciousness. *Front. Psychol.* 2, 4. doi: 10.3389/fpsyg.2011.00004.

Friston, K., Breakspear, M., and Deco, G. (2012). Perception and self-organized instability. *Front. Comput. Neurosci.* 6. doi: 10.3389/fncom.2012.00044.

Friston, K. J. (2019). A free energy principle for a particular physics. *ArXiv190610184 Q-Bio*. Available at: http://arxiv.org/abs/1906.10184 [Accessed July 1, 2019].

Friston, K. J., Kahan, J., Razi, A., Stephan, K. E., and Sporns, O. (2014). On nodes and modes in resting state fMRI. *NeuroImage* 99, 533–547. doi: 10.1016/j.neuroimage.2014.05.056.

Friston, K. J., Parr, T., and de Vries, B. (2017). The graphical brain: Belief propagation and active inference. *Netw. Neurosci.* 1, 381–414. doi: 10.1162/NETN_a_00018.

Friston, K. J., Wiese, W., and Hobson, J. A. (2020). Sentience and the Origins of Consciousness: From Cartesian Duality to Markovian Monism. *Entropy* 22, 516. doi: 10.3390/e22050516.

Garg, N., Vazirani, V. V., and Yannakakis, M. (1996). Approximate max-flow min-(multi) cut theorems and their applications. *SIAM J. Comput.* 25, 235–251.

Ghorbani, A., and Zou, J. (2020). Neuron Shapley: Discovering the Responsible Neurons. *ArXiv200209815 Cs Stat*. Available at: http://arxiv.org/abs/2002.09815 [Accessed December 14, 2020].

Hanson, S. J. (1990). A stochastic version of the delta rule. *Phys. Nonlinear Phenom.* 42, 265–272. doi: 10.1016/0167-2789(90)90081-Y.

Haun, A., and Tononi, G. (2019). Why Does Space Feel the Way it Does? Towards a Principled Account of Spatial Experience. *Entropy* 21, 1160. doi: 10.3390/e21121160.

Heuvel, M. P. van den, and Sporns, O. (2011). Rich-Club Organization of the Human Connectome. *J. Neurosci.* 31, 15775–15786. doi: 10.1523/JNEUROSCI.3539-11.2011.

Hinton, G. (2017). How to do backpropagation in a brain. 22.

Hoffman, A. J. (2003). "A generalization of max flow–min cut," in *Selected Papers Of Alan J Hoffman: With Commentary* (World Scientific), 275–282.

Kachman, T., Owen, J. A., and England, J. L. (2017). Self-Organized Resonance during Search of a Diverse Chemical Space. *Phys. Rev. Lett.* 119, 038001. doi: 10.1103/PhysRevLett.119.038001.

Kaila, V., and Annila, A. (2008). Natural selection for least action. *Proc. R. Soc. Math. Phys. Eng. Sci.* 464, 3055–3070. doi: 10.1098/rspa.2008.0178.

Kingma, D. P., and Welling, M. (2014). Auto-Encoding Variational Bayes. *ArXiv13126114 Cs Stat*. Available at: http://arxiv.org/abs/1312.6114 [Accessed March 29, 2020].

Kinouchi, O., Brochini, L., Costa, A. A., Campos, J. G. F., and Copelli, M. (2019). Stochastic oscillations and dragon king avalanches in self-organized quasi-critical systems. *Sci. Rep.* 9, 1–12. doi: 10.1038/s41598-019-40473-1.

Koller, D., and Friedman, N. (2009). *Probabilistic Graphical Models: Principles and Techniques*. MIT Press.

Kosiorek, A., Sabour, S., Teh, Y. W., and Hinton, G. E. (2019). "Stacked Capsule Autoencoders," in *Advances in Neural Information Processing Systems 32*, eds. H. Wallach, H. Larochelle, A. Beygelzimer, F. d\textquotesingle Alché-Buc, E. Fox, and R. Garnett (Curran Associates, Inc.), 15512–15522. Available at: http://papers.nips.cc/paper/9684-stacked-capsule-autoencoders.pdf [Accessed May 14, 2020].

Kramer, M. A. (1991). Nonlinear principal component analysis using autoassociative neural networks. *AIChE J.* 37, 233–243. doi: 10.1002/aic.690370209.

Lahav, N., Sendiña-Nadal, I., Hens, C., Ksherim, B., Barzel, B., Cohen, R., et al. (2018). Synchronization of chaotic systems: A microscopic description. *Phys. Rev. E* 98, 052204. doi: 10.1103/PhysRevE.98.052204.

Lillicrap, T. P., Santoro, A., Marris, L., Akerman, C. J., and Hinton, G. (2020). Backpropagation and the brain. *Nat. Rev. Neurosci.*, 1–12. doi: 10.1038/s41583-020-0277-3.

Liu, J., Kumar, A., Ba, J., Kiros, J., and Swersky, K. (2019). Graph Normalizing Flows. *ArXiv190513177 Cs Stat*. Available at: http://arxiv.org/abs/1905.13177 [Accessed May 24, 2020].

Madl, T., Baars, B. J., and Franklin, S. (2011). The timing of the cognitive cycle. *PloS One* 6, e14803.

Maschler, M. (1992). The bargaining set, kernel, and nucleolus. *Handb. Game Theory Econ. Appl.* 1, 591–667.

Maschler, M., Peleg, B., and Shapley, L. S. (1979). Geometric properties of the kernel, nucleolus, and related solution concepts. *Math. Oper. Res.* 4, 303–338.

Mayner, W. G. P., Marshall, W., Albantakis, L., Findlay, G., Marchman, R., and Tononi, G. (2018). PyPhi: A toolbox for integrated information theory. *PLOS Comput. Biol.* 14, e1006343. doi: 10.1371/journal.pcbi.1006343.

Mediano, P. A. M., Seth, A. K., and Barrett, A. B. (2019). Measuring Integrated Information: Comparison of Candidate Measures in Theory and Simulation. *Entropy* 21, 17. doi: 10.3390/e21010017.

Mišić, B., Betzel, R. F., Nematzadeh, A., Goñi, J., Griffa, A., Hagmann, P., et al. (2015). Cooperative and Competitive Spreading Dynamics on the Human Connectome. *Neuron* 86, 1518–1529. doi: 10.1016/j.neuron.2015.05.035.

Oliveira, H. M., and Melo, L. V. (2015). Huygens synchronization of two clocks. *Sci. Rep.* 5, 1–12. doi: 10.1038/srep11548.

Palacios, E. R., Isomura, T., Parr, T., and Friston, K. J. (2019). The emergence of synchrony in networks of mutually inferring neurons. *Sci. Rep.* 9, 6412. doi: 10.1038/s41598-019-42821-7.

Parr, T., Markovic, D., Kiebel, S. J., and Friston, K. J. (2019). Neuronal message passing using Mean-field, Bethe, and Marginal approximations. *Sci. Rep.* 9. doi: 10.1038/s41598-018-38246-3.

Safron, A. (2016). What is orgasm? A model of sexual trance and climax via rhythmic entrainment. *Socioaffective Neurosci. Psychol.* 6, 31763.

Safron, A. (2019a). Multilevel evolutionary developmental optimization (MEDO): A theoretical framework for understanding preferences and selection dynamics. *ArXiv191013443 Econ Q-Bio Q-Fin*. Available at: http://arxiv.org/abs/1910.13443 [Accessed November 14, 2019].

Safron, A. (2019b). The radically embodied conscious cybernetic Bayesian brain: Towards explaining the emergence of agency. doi: 10.31234/osf.io/udc42.

Safron, A. (2020). An Integrated World Modeling Theory (IWMT) of Consciousness: Combining Integrated Information and Global Neuronal Workspace Theories With the Free Energy Principle and Active Inference Framework; Toward Solving the Hard Problem and Characterizing Agentic Causation. *Front. Artif. Intell.* 3. doi: 10.3389/frai.2020.00030.

Schmeidler, D. (1969). The nucleolus of a characteristic function game. *SIAM J. Appl. Math.* 17, 1163–1170.

Steppa, C., and Holch, T. L. (2019). HexagDLy—Processing hexagonally sampled data with CNNs in PyTorch. *SoftwareX* 9, 193–198. doi: 10.1016/j.softx.2019.02.010.

Strogatz, S. H. (2012). *Sync: How Order Emerges from Chaos In the Universe, Nature, and Daily Life*. Hachette Books.

Tegmark, M. (2016). Improved Measures of Integrated Information. *PLoS Comput. Biol.* 12. doi: 10.1371/journal.pcbi.1005123.

Toker, D., and Sommer, F. T. (2019). Information integration in large brain networks. *PLOS Comput. Biol.* 15, e1006807. doi: 10.1371/journal.pcbi.1006807.

Whyte, C. J., and Smith, R. (2020). The predictive global neuronal workspace: A formal active inference model of visual consciousness. *Prog. Neurobiol.*, 101918. doi: 10.1016/j.pneurobio.2020.101918.

Willms, A. R., Kitanov, P. M., and Langford, W. F. Huygens' clocks revisited. *R. Soc. Open Sci.* 4, 170777. doi: 10.1098/rsos.170777.
